# Exonic Splicing Mutations Are More Prevalent than Currently Estimated and Can Be Predicted by Using *In Silico* Tools

**DOI:** 10.1371/journal.pgen.1005756

**Published:** 2016-01-13

**Authors:** Omar Soukarieh, Pascaline Gaildrat, Mohamad Hamieh, Aurélie Drouet, Stéphanie Baert-Desurmont, Thierry Frébourg, Mario Tosi, Alexandra Martins

**Affiliations:** 1 Inserm U1079-IRIB, University of Rouen, Normandy Centre for Genomic and Personalized Medicine, Rouen, France; 2 Department of Genetics, University Hospital, Normandy Centre for Genomic and Personalized Medicine, Rouen, France; University Hospital Bonn, GERMANY

## Abstract

The identification of a causal mutation is essential for molecular diagnosis and clinical management of many genetic disorders. However, even if next-generation exome sequencing has greatly improved the detection of nucleotide changes, the biological interpretation of most exonic variants remains challenging. Moreover, particular attention is typically given to protein-coding changes often neglecting the potential impact of exonic variants on RNA splicing. Here, we used the exon 10 of *MLH1*, a gene implicated in hereditary cancer, as a model system to assess the prevalence of RNA splicing mutations among all single-nucleotide variants identified in a given exon. We performed comprehensive minigene assays and analyzed patient’s RNA when available. Our study revealed a staggering number of splicing mutations in *MLH1* exon 10 (77% of the 22 analyzed variants), including mutations directly affecting splice sites and, particularly, mutations altering potential splicing regulatory elements (ESRs). We then used this thoroughly characterized dataset, together with experimental data derived from previous studies on *BRCA1*, *BRCA2*, *CFTR* and *NF1*, to evaluate the predictive power of 3 *in silico* approaches recently described as promising tools for pinpointing ESR-mutations. Our results indicate that ΔtESRseq and ΔHZ_EI_-based approaches not only discriminate which variants affect splicing, but also predict the direction and severity of the induced splicing defects. In contrast, the ΔΨ-based approach did not show a compelling predictive power. Our data indicates that exonic splicing mutations are more prevalent than currently appreciated and that they can now be predicted by using bioinformatics methods. These findings have implications for all genetically-caused diseases.

## Author Summary

The biological interpretation of most disease-associated variants has become a real challenge, especially with the implementation of next-generation sequencing. Particular attention is typically given to protein-coding changes often neglecting the potential impact of exonic variants on RNA splicing. Here, we used the exon 10 of *MLH1*, a gene implicated in hereditary cancer, as a model system to assess the prevalence of RNA splicing mutations among all single-nucleotide variants identified in a given exon by using minigene-based assays. Our study revealed an unexpected high proportion of splicing mutations in *MLH1* exon 10 mostly affecting potential exonic splicing regulatory elements (ESRs), which are typically difficult to predict by using *in silico* tools. We then used five experimental datasets (*MLH1*, *BRCA1*, *BRCA2*, *CFTR* and *NF1*) to evaluate the predictive power of 3 *in silico* approaches recently described for pinpointing ESR-mutations. What’s more, besides predicting which exonic variants affect splicing, ΔtESRseq and ΔHZ_EI_ values also indicated the direction and severity of the induced splicing defects. In contrast, the ΔΨ-based approach did not show a compelling predictive power. Our data indicates that exonic splicing mutations are more prevalent than currently appreciated and that they can now be predicted by using bioinformatics methods. These findings have implications for all genetically-caused diseases.

## Introduction

Tremendous progress has been made in recent years in high-throughput technologies enabling fast and affordable massive parallel DNA sequencing. These methods are now being implemented both in molecular diagnostic settings and in basic research laboratories and hold great promise for discovering the genetic bases of rare and complex diseases [1]. However, even if next-generation sequencing has greatly improved the detection of nucleotide changes in the genome of each individual, the biological and clinical interpretation of most variants remains challenging, representing one of the major hurdles in current medical genetics [2,3]. Several reasons account for the difficulty in distinguishing which variants may cause or contribute to disease [4,5]: (i) the number of single-nucleotide variants (SNVs) detected in each genome is very high, (ii) many disorders are genetically heterogeneous and often caused by rare variants, (iii) access to clinical and family data, such as pedigrees, is frequently limited, (iv) *in silico* tools aiming at identifying deleterious changes are still imperfect, and (v) biological samples from variant carriers are rarely available for functional analysis. There is obviously a great need for overcoming these limitations, and considerable efforts are now deployed for developing strategies allowing prioritization of variants for functional testing [4,6].

In general, scrupulous attention is given to exonic SNVs mapping to protein coding regions, especially to those producing missense changes. Indeed, one of the most widely used strategies for narrowing down the number of variants susceptible of causing disease is to use a combination of *in silico* tools that focus on protein features (such as PolyPhen-2, SIFT and Mutation Assessor, among others) [7–9]. Yet, such protein-centric view of the exome landscape merely represents a fraction of the “expression code” underlying each gene sequence. Current knowledge clearly indicates that, besides their protein coding potential, exonic sequences can play an important role in RNA splicing (reviewed in [10,11]). Notably, (i) the first and the last 3 exonic positions are an integral part of 3’ and 5’splice site (3’ss and 5’ss) consensus sequences, and (ii) exons may also contain splicing regulatory elements (ESRs), such as exonic splicing enhancers (ESEs) and exonic splicing silencers (ESSs). ESEs and ESSs usually correspond to 6–8 nucleotide stretches that serve as landing pads for splicing activator or splicing repressor proteins, respectively, thereby influencing the recruitment and activity of the splicing machinery. Whereas 3’ss and 5’ss consensus sequences have been extensively characterized leading to the development of *in silico* tools that reliably predict alterations in splice site strength (such as MaxEntScan, SpliceSiteFinder-like and Human Splicing Finder, among others), ESRs are still poorly understood and generally regarded as difficult to predict by using bioinformatics approaches [12–15].

Lately, three new *in silico* approaches were described as promising tools to predict variant-induced ESR alterations. The first approach relies on ESRseq scores established through experimental assessment of the ESR properties of all possible 6-nucleotide motifs [16]. Calculation of total ESRseq score changes (ΔtESRseq), taking into account overlapping hexamers and variant-induced changes, was then implemented by our group as a tool to predict the impact on splicing produced by exonic variants [17]. The second approach is based on Z_EI_ scores derived from a RESCUE-type analysis that computed the relative distribution of hexamer motifs in exons and introns [18]. According to this study, the value of total HZ_EI_ score changes (ΔHZ_EI_, also corresponding to overlapping hexamers) can be applied for predicting the impact on splicing of any exonic variant. The third and last approach relies on ΔΨ values (Ψ, percent spliced in) that were bioinformatically established upon compilation of RNAseq data obtained from different tissues, and integration of a large set of pre-established sequence features [19]. The ΔΨ-based approach was developed to predict splicing aberrations induced by any sequence change, either intronic or exonic, including variants affecting splice sites or splicing regulatory signals.

Today, it is estimated that ~15% of all point mutations causing human inherited disorders disrupt splice-site consensus sequences, particularly at intronic positions [20]. Yet, it is now speculated, based on *in silico* data, that disease-causing aberrant RNA splicing may be more widespread than currently appreciated, with up to 25% of exonic disease-associated variants being expected to disturb ESRs [11,21]. Here, we decided to use the exon 10 of the *MLH1* gene as a paradigm to experimentally evaluate these assumptions. *MLH1* exon 10 was selected as model system for 3 main reasons: (i) *MLH1* is the major gene implicated in Lynch syndrome, one of the most frequent forms of hereditary cancer worldwide, formerly known as hereditary nonpolyposis colorectal cancer (HNPCC), (ii) this gene exhibits a large mutational spectrum, with at least 30% of variants being currently classified as variants of unknown significance and for which large national and international efforts persist in bringing clarification [22,23], and (iii) alterations of potential ESRs were already reported for 3 SNVs in this exon [13,24].

We retrieved all SNVs identified in *MLH1* exon 10 and analyzed their impact on splicing by resorting to both minigene-based assays and analysis of patient’s RNA when available. Moreover, we used our experimental data to perform a comparative analysis of the 3 newly developed *in silico* tools aiming at predicting variant-induced ESR alterations. Our results revealed an unexpected high number of splicing mutations in *MLH1* exon 10, most of which affecting potential ESRs, thus corroborating our initial hypotheses. Moreover, we confirmed the predictive power of ΔtESRseq- and ΔHZ_EI_- based approaches, but not that of ΔΨ, for pinpointing this type of mutations.

## Results

### Identification of an unexpected high proportion of splicing mutations in the exon 10 of *MLH1*

**Fig 1:**
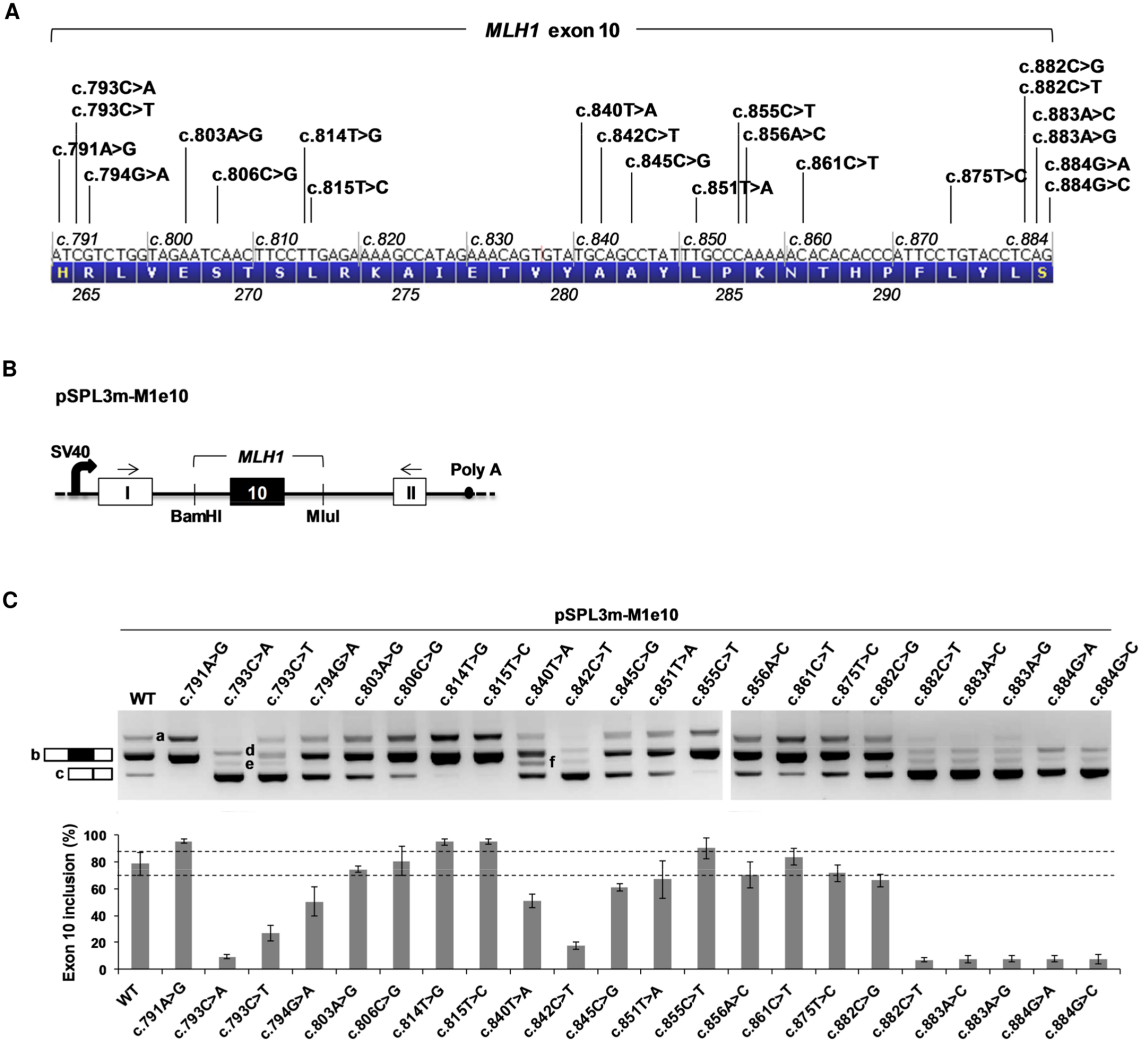
Identification of *MLH1* exon 10 splicing mutations by using a pSPL3m-M1e10 minigene splicing reporter assay. (A) Distribution of all SNVs reported in *MLH1* exon 10 (n = 22). The diagram shows the nucleotide composition of *MLH1* exon 10 (c.791-c.884) and the corresponding amino-acid sequence (1-letter code, p.264-p.295), as well as the position and identity of each SNV. (B) Structure of the pSPL3m-M1e10 minigenes used in the splicing reporter assay. Boxes represent exons and lines in between indicate introns. The minigenes were generated by inserting a genomic fragment containing *MLH1* exon 10 and upstream/downstream flanking intronic sequences (168/187 nucleotides, respectively) into the intron of pSPL3m, as described under Materials and Methods. Arrows above the exons represent RT-PCR primers used in the splicing reporter assay. SV40, SV40 promoter; Poly A, polyadenylation site. (C) Analysis of the splicing pattern of pSPL3m-M1e10 minigenes containing the variants indicated in (A). Wild-type (WT) and mutant pSPL3m-M1e10 constructs were transfected into HeLa cells and then the minigenes’ transcripts were analyzed by RT-PCR as described under Materials and Methods. The top panel shows the RT-PCR products, obtained for WT and mutant constructs as indicated, separated on a 2.5% agarose gel stained with ethidium bromide. The identities of the two major RT-PCR products, with or without exon 10 (b and c, respectively), are indicated on the left. All the products (a, b, c, d, e and f) are described in detail in S1 Fig, f corresponding to RT-PCR products containing exon 10 deleted of the last 48 nucleotides. The bottom panel shows the quantification of the RT-PCR products. Results are shown as the average of three independent experiments and are expressed as percentage of exon inclusion ([exon inclusion products (a+b) x 100/total transcripts]). Error bars indicate individual standard deviation (SD) values, whereas the horizontal dashes delineate the limits of the SD bar obtained for WT (79±9; i.e. 70% and 88% exon inclusion). Variants producing exon inclusion levels outside this range were considered as splicing mutations.

We have recently shown that a high number of variants (15 out of 36, i.e. 42%) identified in the exon 7 of the *BRCA2* gene have a negative impact on splicing [17], a finding strongly suggesting that either *BRCA2* exon 7 is exceptionally sensitive to exonic splicing mutations, or that this type of mutations is more frequent than currently estimated. To test these hypotheses, we decided to study the impact on splicing of SNVs identified in another gene, more specifically in *MLH1*, a gene implicated in Lynch syndrome. Our approach was to use the exon 10 of *MLH1* as a model system. We began by interrogating National and International public databases in order to retrieve all single substitutions reported in this exon (Table 1 and Fig 1A). As a result, we found a total of 22 SNVs, most of which identified in cancer patients suspected of Lynch syndrome, including 15 missense, 3 nonsense and 4 synonymous variants. Only 9 of these SNVs are currently classified as clearly pathogenic, and 1 as clearly not pathogenic (Table 1). To assess the impact of all 22 variants on *MLH1* exon 10 splicing, we performed an *ex vivo* splicing assay with pSPL3m-M1e10-derived minigenes (Fig 1B). As shown on Figs 1C and S1, the wild-type pSPL3m-M1e10 minigene predominantly generated transcripts containing exon 10 (79% exon inclusion), and a minority of transcripts without exon 10. These results are in agreement with those previously reported by using the same minigene system [13], and indicate that wild-type pSPL3m-M1e10 mimics, at least in part, the alternative splicing pattern of endogenous *MLH1* transcripts [25–27] (~35% to 67% exon 10 inclusion in normal blood samples, according to a study from Charbonnier and collaborators [25]). Importantly, the minigene assay results revealed that 17 out of the 22 SNVs (77%) altered the splicing pattern of exon 10 relative to wild-type (Figs 1C and S1). More precisely, 13 variants increased exon skipping, 4 variants increased exon inclusion, and only 5 variants showed no effect on splicing. Of note, 8 of the 13 exon-skipping mutations (5 missense, 1 synonymous, and 2 nonsense) are currently classified as pathogenic (Table 1). The 13 variants increasing exon 10 skipping can be separated into three categories according to the severity of the splicing defect observed in the pSPL3m-M1e10 minigene assay: a first category consisting of 6 variants causing near-total exon skipping (7% to 9% exon inclusion: c.793C>A, c.882C>T, c.883A>C, c.883A>G, c.884G>A and c.884G>C; S1 Table and S3B Fig), a second category consisting of five variants inducing moderate skipping (26% to 67% exon inclusion: c.793C>T, c.794G>A, c.845C>G, c.851T>A and c.882C>G, S1 Table) and a third category consisting of 2 variants (c.840T>A and c.842C>T) that led not only to increased exon 10 skipping (51% and 18% exon inclusion, respectively, S1 Table) but also to deletion of the last 48 nucleotides of the exon in a small fraction of the minigene transcripts (S1B Fig, right panel). A bioionformatics analysis using splice site-dedicated algorithms revealed that both variants, c.840T>A and c.842C>T, create a 5’splice site at exonic position +46 (*MLH1* c.836, S1C Fig), which explains the 48-nucleotide deletion at the end of the exon but not the predominant exclusion of the entire exon.

**Table 1:** *MLH1* exon 10 variants analyzed in this study. *MLH1* exon 10 spans nucleotide positions c.791 to c.884. The first and the last 3 exonic positions (delineated by the dashed line) are an integral part of the 3’ss and 5’ss consensus sequences, respectively. LOVD, Leiden Open Variation Database; dbSNP, the Single Nucleotide Polymorphism database; UMD-MLH1, Universal Mutation Database-MLH1; Swiss-Prot, the European protein sequence database. Variant classification was retrieved from the LOVD database and refers to the 5-tier system used by the InSiGHT Variant Interpretation Committee (http://insight-group.org/variants/classifications/) as follows: 1, not pathogenic; 2, likely not pathogenic; 3, uncertain; (also called VUS for variants of unknown significance); 4, likely pathogenic; 5, pathogenic; n/a, not available.

Position in *MLH1* exon 10	Nucleotide variant	Predicted amino acid change	Databases	Variant classification
+1	c.791A>G	p.His264Arg	LOVD, dbSNP	3
+3	c.793C>A	p.Arg265Ser	LOVD, dbSNP	4
+3	c.793C>T	p.Arg265Cys	LOVD, dbSNP, UMD-MLH1	5
+4	c.794G>A	p.Arg265His	LOVD, dbSNP, UMD-MLH1	3
+13	c.803A>G	p.Glu268Gly	LOVD, dbSNP	1
+16	c.806C>G	p.Ser269*	LOVD, dbSNP, UMD-MLH1	5
+24	c.814T>G	p.Leu272Val	LOVD, dbSNP	3
+25	c.815T>C	p.Leu272Ser	LOVD, dbSNP	n/a
+50	c.840T>A	p.Tyr280*	LOVD, dbSNP	5
+52	c.842C>T	p.Ala281Val	LOVD, dbSNP, UMD-MLH1	5
+55	c.845C>G	p.Ala282Gly	LOVD, dbSNP, Swiss-Prot	2
+61	c.851T>A	p.Leu284*	LOVD, dbSNP, UMD-MLH1	5
+65	c.855C>T	p. = (p.Pro285Pro)	LOVD, dbSNP	n/a
+66	c.856A>C	p.Lys286Gln	LOVD, dbSNP	3
+71	c.861C>T	p. = (p.Asn287Asn)	LOVD, dbSNP	3
+85	c.875T>C	p.Leu292Pro	LOVD, dbSNP, Swiss-Prot	3
+92	c.882C>G	p. = (p.Leu294Leu)	LOVD	3
+92	c.882C>T	p. = (p.Leu294Leu)	LOVD, dbSNP, UMD-MLH1	5
+93	c.883A>C	p.Ser295Arg	LOVD, dbSNP	5
+93	c.883A>G	p.Ser295Gly	LOVD, dbSNP	5
+94	c.884G>A	p.Ser295Asn	LOVD, dbSNP	5
+94	c.884G>C	p.Ser295Thr	LOVD, dbSNP, Swiss-Prot	4

To gain further insight into the severity of the splicing defects of the 13 mutants that induced exon 10 skipping in pSPL3m-M1e10, we tested these variants in the context of another splicing reporter minigene, pCAS2-M1e10 (S2 Fig), previously shown to be less sensitive to splicing mutations than pSPL3m-M1e10 [13]. Our results indicate that, in contrast to pSPL3m-M1e10 and as expected, wild-type pCAS2-M1e10 exclusively generated transcripts containing exon 10. We also observed that 5 out of 6 variants from the first category exhibited a behavior similar to the one observed with the pSPL3m minigenes, i.e. near-total exon 10 skipping; the exception being c.882C>T that induced partial, though strong, exon 10 skipping in the pCAS2 system. Four out of 5 variants from the second category showed splicing defects less severe than the ones observed with pSPL3m-M1e10 and, in one case, c.851T>A, no splicing anomaly was detected. One should note that the level of exon skipping induced by this last variant was borderline noticeable in the pSPL3m-derived minigene (Fig 1C and S1 Table). As for the third category group, we observed that the splicing defects detected in the pSPL3m minigene were faithfully reproduced in pCAS2 for one of the variants (c.842C>T, which predominantly yielded exon-skipped products) but not for the other (c.840T>A). The fact that, in the pCAS2 system, c.840T>A predominantly produced transcripts containing exon 10 deleted of its last 48 nucleotides and almost no exon-skipped products (S2 Fig) suggests that the type and severity of the splicing defect caused by this variant depends on surrounding nucleotide context.

We surmise, given their position at the termini of the exon, that 7 out of the 17 splicing mutations detected in exon 10 directly affect the definition of the reference splice sites, either at the level of the 3’splice site (YAG│G, first exonic position underlined) or of the 5’splice site (CAG│GURAGU, 3 last exonic positions underlined) [10]. Accordingly, the effects produced by these 7 variants (c.791A>G, c.882C>G, c.882C>T, c.883A>C, c.883A>G, c.884G>A and c.884G>C) could have been predicted by algorithms commonly used to predict the strength of splice sites, such as Splice Site Finder, MaxEntScan, and Human Splice Finder (HSF-ss). As shown on S3 Fig, *in silico* data derived from these programs strongly suggest that c.791A>G improves exon inclusion by directly increasing the strength of the 3’splice site, and that the variants at the last three positions of the exon (c.882 to c.884), induce exon skipping by decreasing, to different extents, the strength of the 5’splice site. Moreover, according to this bioinformatics analysis, especially with MaxEntScan (MES), one could have correctly predicted that the splicing defect produced by c.882C>G is less severe than the one induced by c.882C>T. Our data further indicates that a decrease in 5’ss MES score of ≥19% predicts very drastic exon 10 skipping.

Because the 10 remaining splicing mutations detected in *MLH1* exon 10 are located outside the positions directly defining the splice sites, we strongly suspect that they interfere with exon recognition by altering ESRs. This type of splicing mutations includes variants that map within the first two thirds of exon 10 and are responsible for inducing either exon skipping (c.793C>A, c.793C>T, c.794G>A c.840T>A, c.842C>T, c.845C>G and c.851T>A) or exon inclusion (c.814T>G, c.815T>C and c.855C>T).

### Detection of potential splicing regulatory elements in the exon 10 of *MLH1*

**Fig 2:**
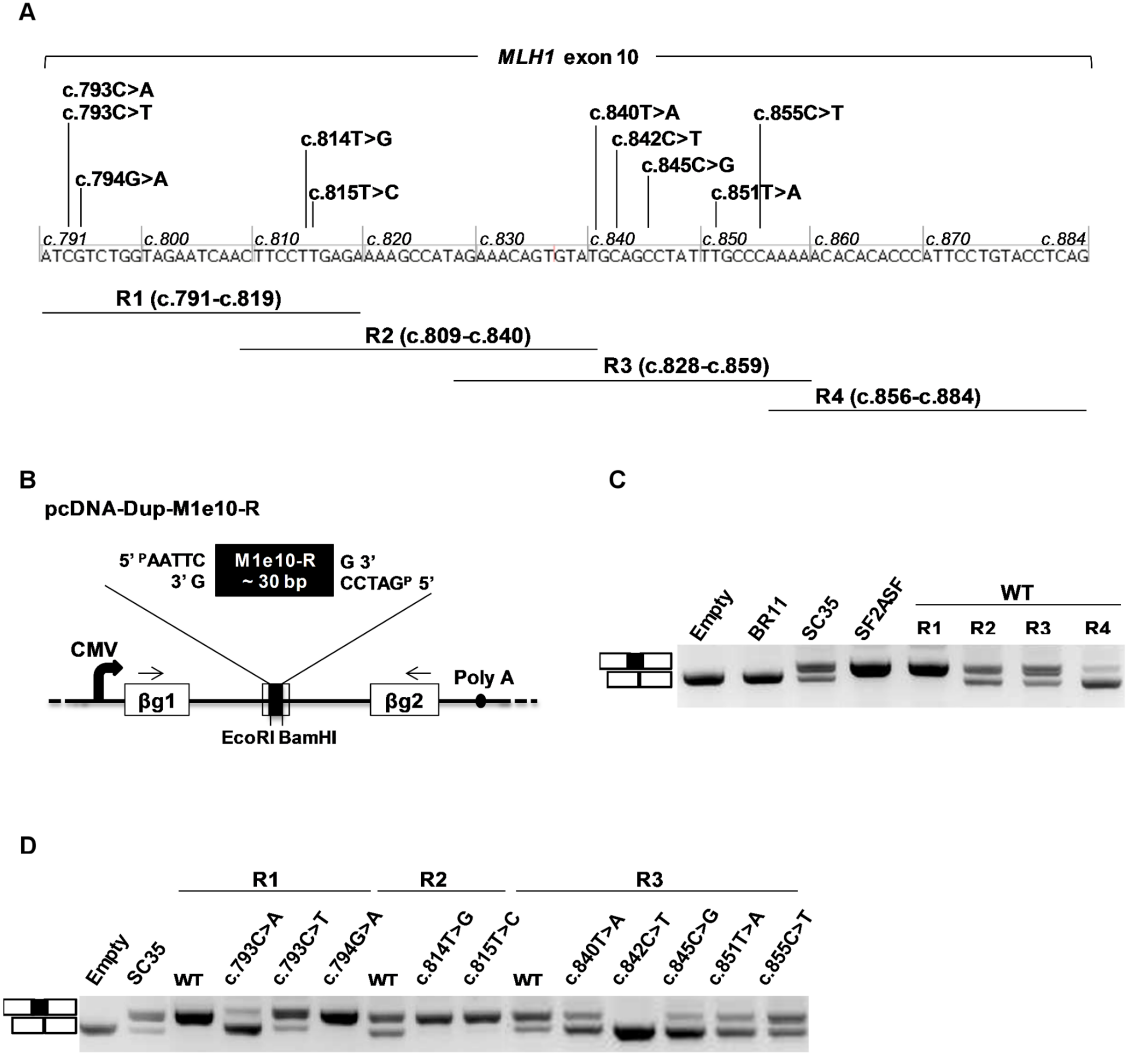
Characterization of *MLH1* exon 10 variants affecting potential ESRs by using an ESR-dependent minigene assay. (A) Strategy for mapping potential splicing regulatory regions in *MLH1* exon 10. The horizontal bars under the sequence of *MLH1* exon 10 represent the ~30 bp-long exonic fragments tested in the ESR-dependent minigene reporter assay (R1 to R4, nucleotide coordinates as indicated). Variants selected for ESR-dependent analysis are indicated above the exon. (B) Structure of the pcDNA-Dup-M1e10-R minigenes used in the ESR-dependent reporter assay. Minigenes were prepared by inserting individual wild-type or mutant *MLH1* exon 10 fragments (M1e10-R as indicated) into the middle exon of pcDNA-Dup, a three-exon vector with a central exon particularly sensitive to ESRs. Boxes indicate exons and lines in between indicate introns. Arrows above the exons represent RT-PCR primers used in the ESR-dependent reporter assay. CMV, CMV promoter; Poly A, polyadenylation site; ßg1 and ßg2, ß-globin exons 1 and 2. (C) Analysis of the splicing pattern of pcDNA-Dup-M1e10-R minigenes containing different wild-type (WT) *MLH1* exon 10 fragments, as indicated. After transfection into HeLa cells the minigenes’ transcripts were analyzed by RT-PCR as described under Materials and Methods. The image shows the RT-PCR products separated on a 2.5% agarose gel stained with ethidium bromide and is representative of 3 independent experiments. The identities of the RT-PCR products are indicated on the left. Empty, BR11, SC35 and SF2ASF refer to previously described negative and positive control pcDNA-Dup minigenes [13]. (D) Comparative analysis of the splicing pattern of pcDNA-Dup-M1e10-R minigenes containing either wild-type or mutant *MLH1* exon 10 fragments, as indicated. The assay was performed as described in (C). The image shows the RT-PCR products separated on a 2.5% agarose gel stained with ethidium bromide and is representative of 3 independent experiments. WT, wild-type.

To better understand how *MLH1* exon 10 splicing is regulated and how certain SNVs disturb this process, we decided to functionally characterize different regions of the exon in the context of pcDNA-Dup. This three-exon minigene contains a middle exon particularly sensitive to alterations of splicing regulatory elements [13,17,28]. For this purpose, four partially overlapping segments covering the entire exon 10 of *MLH1* (R1 to R4, ~30 bp-long each) were individually inserted into the middle exon of this construct and then tested in the context of an *ex vivo* splicing assay (Fig 2A and 2B). As shown in Fig 2C, our results revealed that region R1 (*MLH1* c.791-819) strongly contributed to the recognition of the middle exon. Regions R2 (c.809-c.840), R3 (c.828-c.859) and R4 (c.856-c.884) had a positive effect on exon inclusion as well, although more moderate for R2 and R3 (in comparison to R1), and weak for R4. Next, we tested all *MLH1* exon 10 splicing mutations suspected of altering splicing regulatory elements (n = 10, Fig 2A). Importantly, we found that 8 out of these 10 variations altered the splicing efficiency of the middle exon relative to wild-type (Fig 2D). Variants c.793C>A and c.793C>T (both tested in the context of the R1 segment) induced exon skipping, with c.793C>A having an effect stronger than c.793C>T and thus faithfully recapitulating the defects initially detected in pSPL3m-M1e10 and pCAS2-M1e10 minigene assays. The impact on splicing of variants c.814T>G and c.815T>C was also reproduced in the context of pcDNA-Dup as both variants led to an increase in middle exon inclusion (R2 segment). Finally, variants c.840T>A, c.842C>T, c.845C>G and c.851T>A induced middle exon skipping (R3 segment), with c.842C>T having the more pronounced effect, reminiscent of the effects observed in the pSPL3m-M1e10 and pCAS2-M1e10 assays. Results obtained with c.793C>T and c.842C>T agree with those previously reported by using the pcDNA-Dup system [13]. In contrast, we found that the splicing alterations induced by c.794G>A and c.855C>T in the context of pSPL3m-M1e10 minigene could not be reproduced in the pcDNA-Dup assay. As shown on Fig 2D, c.794G>A did not induce middle exon skipping (R1 segment), and c.855C>T did not lead to an increase in exon inclusion (R3 segment), on the contrary, it slightly increased middle exon skipping. We hypothesize that the effect on splicing of these particular variants depends on surrounding nucleotide context. In conclusion, these experiments point to an apparent asymmetry in the distribution of ESRs in *MLH1* exon 10, with a potential ESE-enrichment at the 5’ portion, corresponding to about the first 2/3 of the exon, and/or an ESS-enrichment towards the 3’end. Moreover, the evidence of portability, i.e. that the splicing defects caused by most of the analyzed SNVs can be reproduced in a completely heterologous system, further supports the hypothesis that these variants modify *bona fide* splicing regulatory elements.

### Bioinformatics approaches based on ΔtESRseq or on ΔHZ_EI_ can predict which exonic variants affect splicing regulatory elements

**Fig 3:**
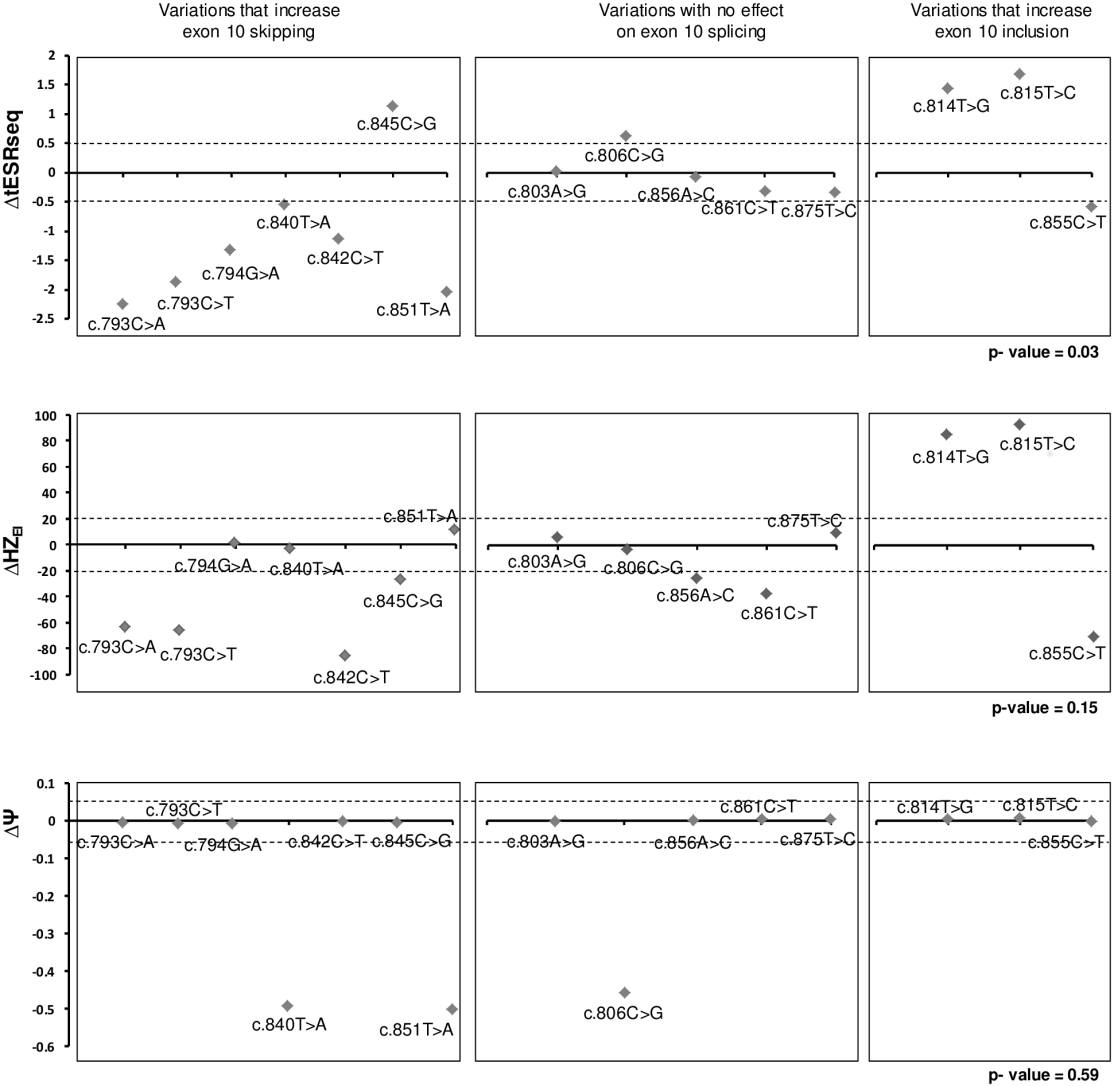
Comparison of pSPL3m-M1e10 minigene data with results obtained from new ESR-dedicated bioinformatics tools. Top, middle and bottom panels refer to results obtained with ΔtESRseq-, ΔHZ_EI-_ and ΔΨ-based bioinformatics approaches, respectively, as described under Materials and Methods. *MLH1* exon 10 variants located within the sequences that define the reference splice sites were eliminated from this analysis. Retained variants were separated into 3 groups depending on their impact on splicing as determined on the pSPL3m-M1e10 minigene assay and indicated above the graphs. Dashed lines indicate the thresholds used in this study. P-values were calculated by using the one-way ANOVA test, as described under Materials and Methods.

Three independent *in silico* approaches based on the calculation of either ΔtESRseq [17], ΔHZ_EI_ [18], or ΔΨ [19] values were recently described as promising tools for predicting variants that potentially modify splicing regulatory elements. Because it remained to be determined if these tools could be applied to new cases and how they compare to each other, we next decided to evaluate their performance by using the output of the pSPL3m-M1e10 minigene assay as a new dataset. We started by separating the fifteen *MLH1* exon 10 SNVs located at distance from the splice sites into three groups based on the minigene results (S1 Table). The first group included mutations that increased exon 10 skipping (n = 7), the second group included variations with no effect on exon 10 splicing (n = 5) and the third group included those that increased exon 10 inclusion (n = 3). Then, score differences (ΔtESRseq, ΔHZ_EI_, and ΔΨ) were calculated for each variant and plotted in a parallel fashion in order to easily confront the discriminating power of the three bioinformatics approaches (Fig 3). Visual inspection of the plots revealed a striking distribution of ΔtESRseq clearly distinguishing the 3 groups of variants. More specifically, most variants with no effect on exon 10 splicing seemed to display ΔtESRseq values higher than those increasing exon skipping and lower than those increasing exon inclusion. This feature was not so clearly noticeable for ΔHZ_EI_ or ΔΨ. A statistical analysis further confirmed that ΔtESRseq values were overall concordant with the separation of variants in 3 groups according to minigene data (ANOVA test, p-value of 0.03), whereas ΔHZ_EI_ and ΔΨ were not (ANOVA test, p-values of 0.15 and 0.59, respectively) (Fig 3 and S1 Table).

To better assess the performances of the three *in silico* approaches, we set preliminary upper and lower thresholds for predicting variant-induced exon skipping and variant-induced exon inclusion: -0.5 and +0.5 for ΔtESRseq, -20 and +20 for ΔHZ_EI_, and -0.05 and +0.05 for ΔΨ [19], respectively (Fig 3). As a consequence, score differences (Δ) smaller than the pre-established negative thresholds were considered indicative of increased exon skipping, whereas those higher than the positive thresholds were considered indicative of increased exon inclusion. We then determined the relative number of true calls produced by each *in silico* approach giving a particular attention to predictions of exon skipping, the most dreaded variant-induced splicing defect. As shown in Fig 3 and S1 Table, we observed that the ΔtESRseq approach produced the highest number of true calls, outperforming ΔHZ_EI_ and ΔΨ in discriminating variants that induce exon skipping from those that do not. More specifically, one can reckon 13 (87%) true calls for ΔtESRseq against 9 (60%) true calls for both ΔHZ_EI_ and ΔΨ. Overall, our results revealed a better sensitivity (86% versus 57%) and specificity (88% versus 63%) for ΔtESRseq than for ΔHZ_EI_ (S1 Table). In spite of displaying a level of specificity similar to ΔtESRseq (88%), ΔΨ showed a very high false negative rate (29% sensitivity). Finally, a statistical analysis further confirmed that ΔtESRseq values could discriminate variants that increased *MLH1* exon 10 skipping from those that did not, whereas ΔHZ_EI_ and ΔΨ could not (t-test, p-values of 0.01, 0.11 and 0.42, respectively).

Results discordant with experimental data, such as the ΔtESRseq values obtained for *MLH1* c.845C>G and c.855C>T (outliers in Fig 3) may be due to features not taken into account by the *in silico* approach such as the RNA secondary structure, the chromatin structure, the presence of ESRs longer than six nucleotides, or to a crosstalk with other splicing regulatory elements located nearby.

**Fig 4:**
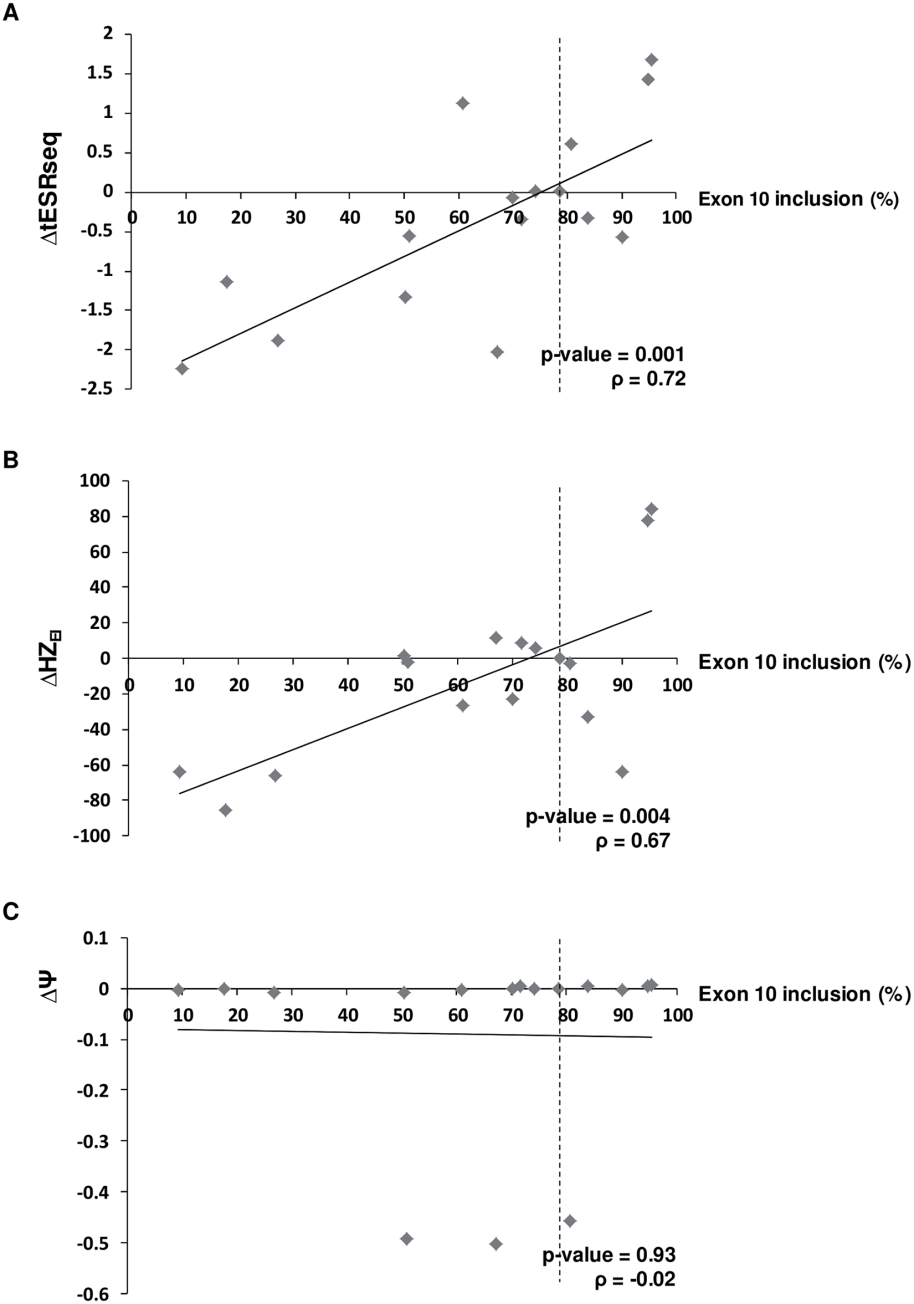
Correlation analysis between exon 10 inclusion levels and results obtained from new ESR-dedicated bioinformatics tools. (A), (B) and (C) refer to results obtained with ΔtESRseq-, ΔHZ_EI-_ and ΔΨ-based bioinformatics approaches, respectively, as described under Materials and Methods. Only *MLH1* exon 10 variants located outside the sequences that define the reference splice sites were retained for this analysis as already mentioned in Fig 3. The precise correspondence between each Δ value (ΔtESRseq, ΔHZ_EI_ or ΔΨ), the level of exon inclusion observed in the pSPL3m-M1e10 minigene assay, and the identity of the corresponding *MLH1* exon 10 variant, is indicated on S1 Table. Correlation coefficients (r) and p-values were determined by performing a Pearson correlation analysis, as described under Materials and Methods.

Next, we wondered if these bioinformatics methods could predict the severity of the splicing defects, i.e. if there was a correlation between the level of exon inclusion detected in the pSPL3m-M1e10 assay and the score differences produced by the ΔtESRseq, ΔHZ_EI_ and ΔΨ approaches. To answer this question, we plotted the minigene data against the score differences generated by the *in silico* approaches, and performed a regression analysis with both variables. As shown on Fig 4 and S1 Table, our results revealed a linear distribution of the *in silico* score differences as a function of exon inclusion levels for ΔtESRseq and ΔHZ_EI_ but not for the ΔΨ approach (Pearson correlation, p-values of 0.001, 0.004 and 0.93, respectively).

Then, we decided to compare the performance of these three new ESR-dedicated *in silico* tools with that of three previously used methods, more precisely EX-SKIP [29], ESEfinder [30,31] and HSF-SR [32]. As shown in S2 Table, EX-SKIP displayed relatively high specificity (75%) but low sensitivity (43%) in predicting exon-skipping mutations. Statistical analyses further revealed that EX-SKIP could not significantly distinguish *MLH1* exon 10 variants that had an effect on splicing from those that did not (t-test and ANOVA test, p-values of 0.22 and 0.26, respectively). Still, we found that there was a correlation between the level of exon 10 inclusion and EX-SKIP values (Pearson correlation, p-value of 0.02). Data derived from ESEfinder, and especially HSF-SR, were difficult to interpret because of the presence of conflicting calls (S2 Table). Moreover, given their lack of comprehensiveness and global quantitation, the performance of these two *in silico* tools could not be statistically analyzed, nor properly compared to that of ΔtESRseq, ΔΔHZ_EI_, ΔΨ or EX-SKIP.

To further evaluate the performance of the recently developed ΔtESRseq, ΔHZ_EI_ and ΔΨ-based approaches, we extended our study to the dataset that first revealed the predictive potential of ΔtESRseq [17]. This dataset includes a total of 32 *BRCA2* exon 7 variants located outside the reference splice sites, some of which cause exon skipping (n = 11, suspected of altering ESRs) and some do not (n = 21), as determined experimentally. As shown in S5 Fig and S3 Table, we found again by using this dataset that ΔtESRseq displayed a slightly better sensitivity and specificity than ΔHZ_EI_ (in this case, 100% versus 91%, and 86% versus 76%, respectively) whereas ΔΨ showed very low sensitivity but high specificity (18% and 94%, respectively). Interestingly, not only ΔtESRseq but also ΔHZ_EI_ could distinguish variants that increased exon skipping from those that did not (t-test, p-values of 3.5 e^-6^ and 5.7 e^-6^, respectively). Again, ΔΨ could not discriminate these 2 groups of variants (t-test, p-value of 0.56). Moreover, we observed a statistically significant correlation between the level of *BRCA2* exon 7 inclusion and the score differences produced by the ΔtESRseq and ΔHZ_EI_ approaches (Pearson correlation, p-values 1.1 e^-6^ and 0.9 e^-3^, respectively), whereas ΔΨ showed no correlation (Pearson correlation, p-value = 0.15) (S6 Fig and S3 Table). Finally, we completed our study by analyzing three additional datasets experimentally characterized by other laboratories, namely: (i) a set of 42 *BRCA1* exon 6 variants [29], (ii) a set of 41 *CFTR* exon 12 variants [33,34] and (iii) a set of 24 *NF1* exon 37 variants [35]. As shown in S4, S5 and S6 Tables, here again, we found that ΔtESRseq and ΔHZ_EI_.had better sensitivity than ΔΨ for predicting which variants induce exon skipping (67–100% and 68–100% versus 0–33% sensitivity, respectively, depending on the dataset). Statistical analyses further highlighted the good performance of ΔtESRseq and especially ΔHZ_EI_, but not that of ΔΨ for discriminating variants that lead to exon skipping and for predicting the severivity of the splicing defect within these 3 additional datasets (S7 Table).

We surmise that out of the three new *in silico* approaches expected to predict ESR-mutations [17–19], ΔtESRseq and ΔHZ_EI_ show the best performance at least for the five datasets analyzed in our study. Indeed, these two approaches displayed a better balance between sensitivity and specificity than ΔΨ, or the prior *in silico* method EX-SKIP, for predicting exon skipping-mutations (S8 Table). Importantly, we found that ΔtESRseq and ΔHZ_EI_ can be used to predict not only the direction but also the severity of the induced splicing defects, more negative score differences being indicative of higher exon skipping levels (S7 Table).

### *MLH1* c.793C>T and c.842C>T are associated with drastic splicing defects in patients’ cells

To evaluate the physiological pertinence of the *MLH1* exon 10 minigene assays and the above described *in silico* predictions, we set to compare our results with data derived from the analysis of RNA obtained from carriers of *MLH1* exon 10 variants, especially of those located outside splice sites. Patients’ RNA being rarely available, we had the opportunity to obtain patient RNA samples for only two SNVs of interest: (i) *MLH1* c.793C>T (Patient P_793CT.1_) and (ii) *MLH1* c.842C>T (Patient P_842CT.1_).

**Fig 5:**
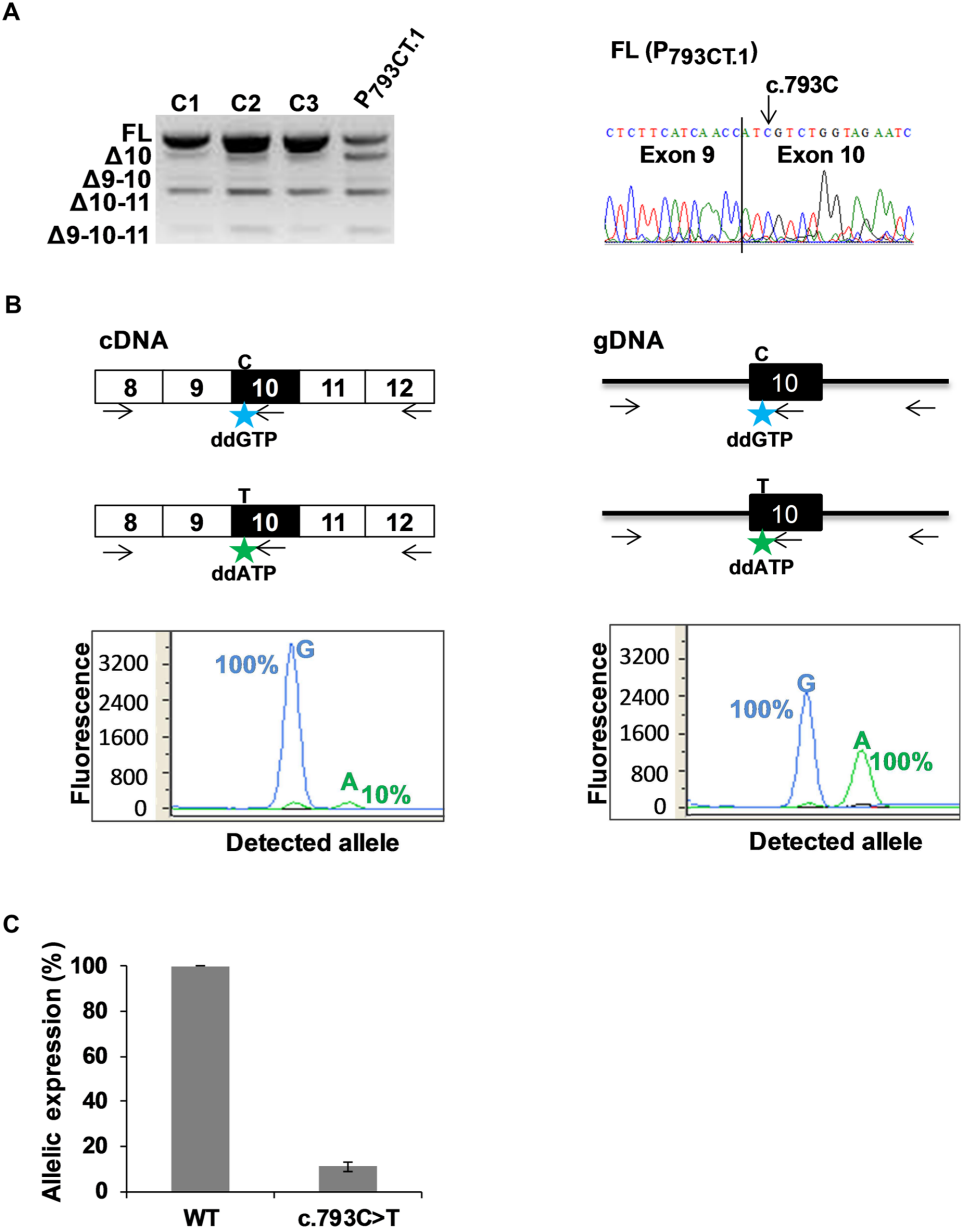
Detection of aberrant splicing in the blood cells of a patient carrying *MLH1* c.793C>T variant. (A) Comparative RT-PCR analysis of fresh blood RNA samples obtained from 3 healthy control individuals (C1, C2 and C3) and from a patient carrying the heterozygous variant *MLH1* c.793C>T (P_793CT.1_). RT-PCR reactions were performed with primers mapping to *MLH1* exon 8 (forward primer) and exon 12 (reverse primer), as described under Materials and Methods. The panel on the left shows the RT-PCR products separated on a 2% agarose gel, and is representative of 3 independent experiments. RT-PCR product identifiers are indicated on the left of the gel. The sequence on the right refers to the FL product obtained from patient P_793CT.1_, with a vertical line indicating the junction between exons 9 and 10. FL, full-length; Δ, exon skipping. (B) Allele-specific expression of *MLH1* in the blood cells of patient P_793CT.1_ carrying the heterozygous variant *MLH1* c.793C>T. PCR, RT-PCR and primer extension reactions were performed as described under Materials and Methods, and are schematically represented above the graphs. The discriminating nucleotides C and T are indicated. Boxes represent exons, lines indicate intronic sequences, and arrows symbolize reaction primers. The discriminating fluorophore-labeled ddG and ddA terminators, incorporated into the noncoding strand at the c.793 position, are represented by stars. Results from complementary DNA (cDNA) analysis and genomic DNA (gDNA) are shown on the left-hand and on the right-hand graphs, respectively. Results (peak areas) obtained with cDNA were normalized to those obtained with gDNA and are expressed, in the left-hand graph, as relative level (in percentage) of FL transcripts produced by each *MLH1* allele. (C) Relative contribution of each allele to the expression of full-length *MLH1* transcripts in the blood cells of patient P_793CT.1_ carrying the heterozygous variant *MLH1* c.793C>T. The graph displays the expression level of the mutant allele (*MLH1* c.793C>T) relative to wild-type (WT). Results are shown as the average of 3 independent experiments performed as described in (B). Error bars indicate standard deviation values.

First, peripheral blood RNA of patient P_793CT.1_ (heterozygous for *MLH1* c.793C>T) was analyzed by RT-PCR, by using primers targeting exons 8 and 12, and compared to those of three control individuals. Our results revealed a complex splicing pattern involving *MLH1* exons 9, 10 and 11 in all individuals (Figs 5A and S4). These data are concordant with previous studies describing exon 10 as an alternative exon that is naturally partially skipped, either alone or in combination with exon 9 and/or exon 11 [25–27]. We also observed that, as compared to controls, the sample derived from patient P_793CT.1_ showed a lower amount of full-length transcripts (FL), and a higher amount of transcripts lacking exon 10 (Δ10), indicative of aberrant splicing. Sequencing of the FL products revealed the presence of WT (c.793C) exon 10 only (Fig 5A, right panel), which suggests that in this sample the exon 10-skipped products mostly derive from the mutant allele (c.793T), and that the variant-associated splicing defect is very severe. Importantly, the observation that c.793C>T is associated with a drastic splicing defect in endogenous *MLH1* transcripts agrees with the data obtained in the minigene assays (Figs 1, 2 and S2), especially in the context of pSPL3m-M1e10 c.793C>T, which showed a very high level of exon skipping. To better evaluate the consequences of c.793C>T on *MLH1* expression, and because Sanger sequencing is known to have low detection sensitivity, we then decided to measure the relative contribution of the WT and mutant alleles to the production of the full-length (FL) transcripts by using an allele-specific primer extension method. Our findings, shown on Fig 5B and 5C, indicate that the FL transcripts expressed from the mutant allele were in fact present in the blood cells of patient P_793CT.1_ but at very low level as compared to the WT allele (~10%). Given the results of our minigene assays, and RT-PCR analysis of patient’s RNA, we conclude that this allelic imbalance is mostly due to c.793C>T-induced exon 10 skipping. Moreover, if one assumes that, in the blood cells of Patient P_793CT.1_, both alleles are transcribed in equal amounts, then one can deduce that the pSPL3m-M1e10 assay closely reflects the effects detected *in vivo* in patient’s peripheral blood, at least for this variant.

We then analyzed LCL RNA from patient P_842CT.1_ (heterozygous for *MLH1* c.842C>T) by comparing to those from 5 controls, including: 3 healthy individuals, and 2 Lynch syndrome patients (P_791-5TG.1_ and P_882CT.1_) carrying *MLH1* c.791-5T>G and c.882C>T mutations directly altering the 3’ss or 5’ss of exon 10, respectively [36]. Results shown on S7 Fig indicate that patient P_842CT.1_ has a splicing pattern similar to that of patients P_791-5TG.1_ and P_882CT.1_, i.e. an apparent decrease in the amount of FL *MLH1* transcripts, a mild increase in exon 9–10 skipping (Δ9–10) and a drastic increase in exon 10 skipping (Δ10), as compared to healthy controls. Δ10 transcripts were detected in LCLs treated with puromycin, strongly suggesting that the aberrant Δ10 out-of-frame transcripts (S4B Fig) are degraded by the NMD pathway in these cell lines. As expected, the level of Δ9–10 in-frame transcripts did not increase in the presence of the NMD-inhibitor puromycin. Sequencing of the FL RT-PCR products of patients P_842CT.1_ and P_882CT.1_ revealed the absence of c.842T and c.882T mutant FL transcripts (S7C Fig), indicating that c.842C>T and c.882C>T cause very severe splicing defects. These *in vivo* results clearly agree with the pSPL3m and pCAS2 minigene assays, which revealed predominant exon 10 skipping for both c.842C>T and c.882C>T (Figs 1, 2 and S2).

Importantly, these results highlight the physiological pertinence of the *in silico* predictions produced by the ΔtESRseq and ΔHZ_EI_ approaches. Indeed, these methods accurately predicted the variant-induced splicing aberrations observed *in vivo* in patients carrying exonic SNVs, as shown here for *MLH1* variants c.793C>T and c.842C>T, and (ii) *BRCA2* c.520C>T or c.617C>G [17,37]. As of note, the ΔtESRseq approach also accurately predicted the physiological effect of *MLH1* c.794G>A [13]. We conclude that ΔtESRseq and ΔHZ_EI_, but not ΔΨ, are promising tools for prioritizing exonic variants for splicing assays.

## Discussion

The present study was initiated to follow up on our observation that a large number of variants in the exon 7 of *BRCA2* induce exon skipping [17]. Our hypothesis was that exonic splicing mutations were also underestimated in other exons and genes. We thus decided to analyze the impact on splicing of all SNVs identified in the exon 10 of *MLH1* (n = 22), a gene implicated in Lynch syndrome. Before this study, only 5 SNVs in *MLH1* exon 10 had been reported as causing aberrant splicing; more specifically, they were all shown to increase exon 10 skipping [13,24,36]. Our work not only confirmed those initial findings but, importantly, uncovered 12 new splicing mutations (8 exon skipping-, and 4 exon inclusion-mutations), bringing the number of *MLH1* exon 10 splicing mutations to a total of 17. Hence, our results revealed a striking high proportion of splicing mutations in *MLH1* exon 10 (77%), largely exceeding the fraction of splicing mutations detected in *BRCA2* exon 7 (42%) [17]. Moreover, we found that the majority of *MLH1* exon 10 splicing mutations (~60%) map outside the reference splice-site consensus sequences, indicating an important contribution of variant-induced ESR alterations in this exon.

Importantly, among the 12 new splicing mutations in *MLH1* exon 10, we identified 4 SNVs causing increased exon inclusion. To our knowledge, this is the first report of SNVs having a positive impact on *MLH1* splicing. Besides full-length transcripts, *MLH1* is known to normally produce a fraction of transcripts lacking exon 10, such as Δ10, Δ9/10, Δ10/11 and Δ9/10/11 [25,26], with Δ9/10 being one of the most frequently reported alternative *MLH1* isoforms [27]. It is possible that the 4 variants that increased exon 10 inclusion in our minigene assays also disturb *MLH1* physiological alternative splicing leading to a higher production of FL transcripts and lower amount of Δ10, Δ9/10 and Δ9/10/11. Because the role of alternative *MLH1* isoforms is still unknown [27], it is difficult to predict the biological and clinical consequences of these splicing alterations. Of note, a few cases of variant-induced exon inclusion have already been described in other genes. Particularly, previous studies have shown that mutations that increase exon inclusion can have a significant clinical impact. For instance, they can behave as protective factors, as is the case of *SMN2* c.859G>C (p.Gly287Arg), a variant that attenuates the severity of spinal muscular atrophy (SMA) by increasing inclusion of *SMN2* exon 7 [28,38]. Moreover, mutations inducing exon inclusion can also be harmful, as is the case for the majority of mutations identified in the exon 10 of the *Microtubule Associated Protein Tau* gene (*MAPT*) (reviewed in [39]). It has been shown that dysregulation of *MAPT* exon 10 splicing disrupts normal tau isoform ratio and leads to neurodegeneration and dementia: increased *MAPT* exon 10 skipping causes Pick disease, whereas increased inclusion typically causes FTDP-17 (frontotemporal dementia with parkinsonism linked to chromosome 17).

Given the challenging need in medical genetics for stratifying exonic variants for functional analyses, we decided to use the experimental data generated in this study to evaluate the predictive power of *in silico* tools at discerning splicing mutations. We found that the impact on splicing of the seven *MLH1* exon 10 variants mapping within the splice-site consensus sequences (potential splice site-mutations) were correctly predicted by splice site-dedicated *in silico* tools (SSF, MES and HSF-ss). These findings confirm and extend previous studies that highlighted the good reliability of these algorithms for predicting exon skipping-mutations [12–15], further pinpointing their interest as filtering tools in variant stratification strategies. As for the variants located outside the reference splice sites, our minigene data revealed 10 ESR-mutations and 5 variants with no impact on splicing. We took advantage of these results and selected four additional experimental datasets previously described in other genes [17,29,33–35] to evaluate the discriminating power of 3 bioinformatics approaches recently described as suitable for predicting variant-induced ESR alterations [16–19]. Our findings revealed that the ΔtESRseq and ΔHZ_EI_ ESR-dedicated tools show the best performance in identifying ESR-mutations, outperforming the previous bioinformatics method EX-SKIP, whereas ΔΨ did not show compelling predictive power. Our results further indicate that both ΔtESRseq- and ΔHZ_EI-_ based approaches can predict the severity of the variant-induced splicing defects, underlining the quantitative character of these methods. It is possible that the ΔtESRseq- and ΔHZ_EI-_based approaches produced somewhat similar results because they both rely on the appreciation of hexamer sequences as ESRs. Interestingly, a correlation between ESRseq and Z_EI_ scores has already been reported [18] suggesting that most ESRs are indeed defined by 6-nucleotide stretches and that ESE sequences are more frequently represented in exons than in introns. Contrary to our initial expectations, ΔΨ displayed the weakest predictive power out of the 3 new *in silico* tools dedicated to identifying ESR alterations. This was unexpected as this method had been validated by using a large set of previously published splicing data, such as data on a plethora of *MLH1* nucleotide variants [19]. Close inspection of the validation set used on that study revealed a large excess of intronic variants relative to exonic changes, and also a considerable under-representation of known ESR-mutations relative to the total number of variants included in the dataset. Most of the positive predictions reported for ΔΨ [19] thus correspond to intronic mutations directly affecting splice sites. This may explain why we detected an excess of true negative calls relative to true positive calls when using ΔΨ-values for predicting ESR-mutations in the five datasets analyzed in this study, and suggests that the ΔΨ-based method may be overall suitable for predicting mutations directly modifying splice sites but not entirely reliable for predicting those affecting ESRs. In sum, our findings suggest that both ΔtESRseq- and ΔHZ_EI-_, based approaches can be used to stratify exonic variants for functional testing, and that this strategy may help identifying disease-causing variants. We cannot exclude that ΔΨ-values may be useful in particular conditions and, conversely, that the ΔtESRseq- and ΔHZ_EI-_, based approaches may not be suitable for the analysis of certain exons or genes.

In the case of *MLH1*, it is clear that severe exon 10 skipping causes Lynch syndrome (reviewed in [27]). Skipping of *MLH1* exon 10 leads to a shifted reading frame, resulting in a premature stop codon in exon 11 (MLH1 p.His264Leufs*2) and probable degradation of the aberrant transcripts by NMD. Variants inducing total exon 10 skipping cause a drastic loss in FL MLH1 protein and are therefore considered deleterious. In contrast, the clinical significance of variants inducing partial exon 10 skipping is still unknown. First, it is unclear if the amount of FL transcripts produced in the presence of such variants is enough to fulfill *MLH1* function. Second, it is possible that remaining FL *MLH1* transcripts carrying missense variants lead to production of nonfunctional proteins. Additional analyses are thus necessary to determine the biological and clinical significance of partial exon skipping-variants, including protein assays, assessment of patient clinical history and family data. Given that exon 10 codes for part of an important domain of the MLH1 protein (interaction with MUTSα) [40], we suspect that SNVs increasing exon 10 inclusion can either have a protective impact if co-occurring with variants showing the opposite effect, or a deleterious effect if introducing a missense change that severely impairs protein function. Thus, in the absence of further information, *MLH1* missense SNVs inducing exon 10 inclusion, as well as those not affecting splicing, should be considered as variants of unknown significance (VUS). In contrast, the clinical classification of synonymous substitutions not affecting RNA splicing can eventually evolve from VUS to likely not pathogenic depending on expert panel assessment [22,23].

In conclusion, our results revealed an unexpected high number of splicing mutations in the exon 10 of *MLH1*, most of which affecting potential ESRs, and confirmed the predictive power of ΔtESRseq- and ΔHZ_EI_-based approaches for pinpointing this type of mutations, at least in *MLH1* exon 10, *BRCA2* exon 7, *BRCA1* exon 6, *CFTR* exon 12 and *NF1* exon 37. In principle, the bioinformatics methods described in our study are amenable to automation and, as such, have the potential to be used as filtering tools for identifying disease-causing candidates among the large number of variants detected by high-throughput DNA sequencing.

## Materials and Methods

### Ethics statement

Written informed consent was obtained from all individuals.

### Retrieval of *MLH1* exon 10 variants

We collected all SNVs reported in the exon 10 of *MLH1*, until January 2013, by interrogating the following public databases: UMD-MLH1 (Universal Mutation Database-MLH1, http://www.umd.be/MLH1/) [22], LOVD (Leiden Open Variation Database, http://chromium.liacs.nl/LOVD2/colon_cancer/variants.php?select_db=MLH1), dbSNP (the Single Nucleotide Polymorphism database http://www.ncbi.nlm.nih.gov/SNP/), and UniProtKB/Swiss-Prot (the European protein sequence database, http://swissvar.expasy.org/cgi-bin/swissvar/result?global_textfield=MLH1) (Table 1 and Fig 1A).

### Nomenclature

Nucleotide numbering is based on the cDNA sequence of *MLH1* (NCBI accession number NM_000249.3), c.1 denoting the first nucleotide of the translation initiation codon, as recommended by the Human Genome Variation Society.

### Splicing minigene reporter assays

In order to evaluate the impact on splicing of each *MLH1* exon 10 variant, we performed functional assays based on the comparative analysis of the splicing pattern of wild-type and mutant MLH1 reporter minigenes. These minigenes were prepared by using two different vectors: pSPL3m and pCAS2. The pSPL3m plasmid, a modified version of the exon-trapping vector pSPL3 (Invitrogen) which in turn derives from pSPL1 [41], carries two chimeric exons (here named I and II, both containing rabbit *β-globin* and HIV *Tat* sequences) separated by an intron containing BamHI and MluI cloning sites (Fig 1B) [13]. Expression of the pSPL3m minigene is driven by the SV40 promoter. The pCAS2 vector carries two exons (here named A and B) with a sequence derived from the human *SERPING1/C1NH* gene, separated by an intron with BamHI and MluI cloning sites (S2A Fig). Expression of the pCAS2 minigene is under the control of a CMV promoter. The pCAS2 is a modified version of the previously described pCAS1 plasmid [13,42]. Two modifications were introduced into the exon A of pCAS2 relative to pCAS1: (i) the first 114 bp of exon A were deleted, and (ii) the *SERPING1*/*CINH* translation initiation codon was disrupted by replacing the sequence GATG (initiation codon underlined) by TCAC.

The wild-type genomic fragment *MLH1* c.791-168_c.884+187 (*MLH1* exon 10 and flanking intronic sequences) was inserted into the BamHI and MluI cloning sites of the reporter plasmids pSPL3m and pCAS2, yielding the three-exon hybrid minigenes pSPL3m-M1e10 and pCAS2-M1e10, respectively (Figs 1B and S2A). Minigenes carrying *MLH1* exon 10 variants were prepared by site-directed mutagenesis by using the two-stage overlap extension PCR method [43] and the wild-type pSPL3m-M1e10 construct as template. Then, the mutant amplicons were digested with BamHI and MluI, and introduced into BamHI and MluI cloning sites of the pSPL3m-M1e10 minigene to replace the wild-type fragment. The inserts of all constructs were sequenced to ensure that no other mutations had been introduced during the cloning process. In some cases, as indicated (S2 Fig), mutant inserts were digested from the pSPL3m-M1e10 minigene with BamHI and MluI and then subcloned into pCAS2.

Next, wild-type and mutant minigenes (1μg/well) were transfected in parallel into HeLa cells grown at ~60% confluence in 6-well plates using the FuGENE 6 transfection reagent (Roche Applied Science). HeLa cells were cultivated in Dulbecco’s modified Eagle medium (Life Technologies) supplemented with 10% fetal calf serum in a 5% CO2 atmosphere at 37°C. Total RNA was extracted 24 hours after transfection using the NucleoSpin RNA II kit (Macherey Nagel) according to the manufacturer’s instructions. Then, the minigene transcripts were analyzed by semi-quantitative RT-PCR (30 cycles of amplification) in a 25 μl reaction volume by using the OneStep RT-PCR kit (Qiagen), 100 ng total RNA, and pSPL3m- or pCAS2-appropriate forward and reverse primers (SD6 and SA2 or pCAS-KO1-F and pCAS-2-R, respectively, as described in [13] and [37]). RT-PCR products were separated by electrophoresis on 2.5% agarose gels containing ethidium bromide and visualized by exposure to ultraviolet light under non-saturating conditions using the Gel Doc XR image acquisition system (Bio-RAD). Semi-quantitative analysis, gel extraction and sequencing of the RT-PCR products were carried out as previously described [42].

### ESR-dependent minigene reporter assay

*MLH1* exon 10 fragments (~30 bp-long) were analyzed for their splicing enhancer properties by performing a functional assay based on the splicing pattern of the pcDNA-Dup minigene [13]. This vector contains a β-globin-derived three-exon minigene with a middle exon particularly sensitive to the presence of exonic splicing regulatory signals. Expression of the minigene is under the control of the CMV promoter (Fig 2B). The exonic fragments of interest, wild-type or mutant, were obtained by annealing complementary 5’-phosphorylated oligonucleotides carrying 5’-EcoRI and 3’-BamHI compatible ends. Then, the exonic segments were inserted into the EcoRI and BamHI cloning sites of the middle exon of pcDNA-Dup to produce hybrid pcDNA-Dup-M1e10-R minigenes. All constructs were sequenced to ensure that no other mutations were introduced into the middle exon during the cloning process. Transfection, RNA extraction and RT-PCR analysis were performed as described for the splicing minigene reporter assay except that RT-PCR reactions were performed with 150 ng total RNA as template, T7-Pro (5’-TAATACGACTCACTATAGG-3’) and Dup-S4-Seq-3R (5’-CGTGCAGCTTGTCACAGTGC-3’) as forward and reverse primers respectively, and 28 cycles of amplification. RT-PCR products were analyzed by electrophoresis as described above for the splicing minigene reporter assay.

### Bioinformatics predictions of splice site-mutations

Three *in silico* tools were used to predict variant-induced alterations in 3’ and 5’ splice site strength, namely: SpliceSiteFinder-like (SSF, http://www.interactive-biosoftware.com), MaxEntScan (MES, http://genes.mit.edu/burgelab/maxent/Xmaxentscan_scoreseq.html; Maximum Entropy Model) and the splice site module of Human Splicing Finder (here named HSF-ss for splice site-dedicated HSF, http://www.umd.be/HSF/). These algorithms were interrogated simultaneously by using the integrated software tool Alamut (Interactive Biosoftware, France, http://www.interactive-biosoftware.com), as previously described [17].

### Bioinformatics predictions of ESR-mutations

Three newly developed *in silico* approaches were used to predict variant-induced alterations in exonic splicing regulatory elements (ESRs): (i) calculation of total ESRseq score changes (ΔtESRseq) by using the method previously described by our group [17] with a small modification (here, only exonic positions were taken into account), (ii) calculation of ΔHZ_EI_ values by using the HEXplorer method [18], and (iii) assignment of ΔΨ values based on the Splicing Regulatory Model (http://tools.genes.toronto.edu) recently described by Xiong and co-workers [19]. Moreover, as indicated, we also resorted to three previously established ESR-dedicated *in silico* tools: (i) EX-SKIP (http://ex-skip.img.cas.cz/) [29] in which we took into account the full nucleotide sequence of the exon of interest, (ii) ESEfinder (http://rulai.cshl.edu/cgi-bin/tools/ESE3/esefinder.cgi?process=home) [30,31], and (iii) the ESR module of Human Splicing Finder (here named HSF-SR for ESR-dedicated HSF, http://www.umd.be/HSF3/) [32].

### Statistical analysis

Results are presented as the mean ± SD of three independent experiments. Data derived from comparisons of experimental and *in silico* analyses were compared by using either the one-way ANOVA test or the Student’s t-test, and the Pearson’s correlation coefficient, as indicated. More specifically, the ANOVA test was used for assessing the performance of the bioinformatics tools in discriminating 3 groups of variants (i.e. variants that increase exon skipping versus those with no effect on splicing versus those that increase exon inclusion), whereas Student’s t-test was used when only 2 groups of variants were taken into account (i.e. variants that increase exon skipping versus those that do not). Correlation between exon inclusion levels and *in silico* predictions was measured by calculating Pearson correlation coefficients (r). p-values and r are indicated in the figures. Results were considered significant when p-value <0.05. Statistical tests were performed by using BiostaTGV (http://marne.u707.jussieu.fr/biostatgv/).

The power to distinguish mutations that induce exon skipping from those that do not was further assessed, for each ESR-dedicated *in silico* method, by calculating sensitivity and specificity values (true positives x 100/(true positives + false negatives) and (true negatives x 100/(true negatives + false positives), respectively). Sensitivity and specificity were determined by taking into account the following thresholds: -0.5 for ΔtESRseq (arbitrary threshold), -20 for ΔHZ_EI_ (arbitrary threshold), and -0.05 for ΔΨ (threshold previously established by the authors, [19].

### Analysis of the splicing pattern of *MLH1* in peripheral blood samples and lymphoblastoid cell lines of patients and controls

Peripheral blood samples were directly collected into PAXgene Blood RNA Tubes (Qiagen) from which total RNA was extracted by using the PAXgene Blood RNA kit, according to the manufacturer’s instructions. EBV-immortalized lymphoblastoid cell lines (LCLs) were cultivated in RPMI medium (Life Technologies) supplemented with 2 mM of L-glutamine and 10% fetal calf serum, at 37°C in a 5% CO2 atmosphere. Before RNA extraction, LCLs were transferred into 6-well plates, at 2.5x10^6^ cells/well, and incubated for 5.5 hours with/without 200 μg/ml puromycin. Then, total RNA was extracted by using the NucleoSpin RNA II kit (Macherey Nagel). Written informed consent was obtained from all individuals.

The splicing pattern of *MLH1* transcripts expressed in peripheral blood and in LCLs was analyzed by semi-quantitative RT-PCR using the OneStep RT-PCR kit (Qiagen) in 25 μl-final volume reactions containing 100 ng of total RNA, a forward primer located in *MLH1* exon 8 (MLH1-RT-8Fbis, 5’-AAGGAGAGACAGTAGCTGATGTT-3’) and a reverse primer located in exon 12 (MLH1-12R, 5’-TGCTCAGAGGCTGCAGAAA-3’). To ensure that the assay was in the linear range, RT-PCR reactions were performed with 34 cycles of amplification (S4 Fig). Then, RT-PCR products were separated by electrophoresis on a 2% agarose gel, gel-purified and sequenced.

### Allele specific expression analysis

Allele specific expression (ASE) was measured by performing a SNaPshot assay (ABI Prism SNaPshot, Fig 5B). First, RT-PCR products spanning *MLH1* exons 8 to 12 were obtained, as described above, from a peripheral blood RNA sample of a patient carrying the heterozygous c.793C>T substitution in *MLH1* exon 10 (Patient P_793CT.1_). In parallel, a segment encompassing *MLH1* exon 10 was amplified by PCR from the genomic DNA of the same patient by using the Multiplex PCR kit (Qiagen) according to the manufacturer’s instructions. Briefly, PCR reactions (35 cycles of amplification) were performed in a final volume of 25 μl with 100 ng of genomic DNA as template, a forward primer in *MLH1* intron 9 (MLH1-10-Bam-F, 5’-GACCGGATCCTTGGAAAGTGGCGACAGG-3’) and a reverse primer in intron 10 (MLH1-10-Mlu-R, 5’-GACCACGCGTAATTAGTGAATAAATGAAGGAAAA-3’). Then, 5 μl aliquots of RT-PCR and PCR products were treated with one unit of Shrimp Alkaline Phosphatase (SAP, USB) and 8 units of Exonuclease I (Thermo Scientific) in the presence of SAP buffer in a final volume of 10 μl. After 1 hour at 37°C, the reactions were terminated by incubating at 75°C for 15 minutes. Next, 2 μl aliquots of treated RT-PCR and PCR products were subjected to SNaPshot reactions, in a final volume of 10 μl, by using the SNaPshot Multiplex Kit (Applied Biosystems) and a reverse primer targeting the sequence immediately downstream *MLH1* c.793C>T (SNAP-M1.793-R, 5’-GGAAGTTGATTCTACCAGAC-3’). SNaPshot reactions were carried out by performing 25 cycles of primer extension (denaturation at 96°C for 10 sec, annealing at 50°C for 5 sec and elongation at 60°C for 30 sec). Next, the reactions were incubated with 1 unit of SAP at 37°C for 1 hour, and terminated at 75°C for 15 minutes. Finally, the extension products were separated by electrophoresis and analyzed quantitatively by using an ABI PRISM-3100 Genetic Analyzer (Applied Biosystems). SNaPshot results obtained from patient cDNA were normalized by those obtained from gDNA.

## Supporting Information

S1 Fig Description of the RT-PCR products obtained in the pSPL3m-M1e10 minigene splicing reporter assay. (A) Comparative RT-PCR analysis of the splicing patterns of wild-type and mutant pSPL3m-M1e10 minigenes, as indicated. The image shows the RT-PCR products separated on a 2.5% agarose gel as described in Fig 1C. (B) Splicing events underlying the production of the major (a, b and c) and minor (d, e and f) RT-PCR products visualized in (A). As indicated, RT-PCR products were separated into 3 groups according to the splicing behavior of *MLH1* exon 10. Boxes represent exons and lines in between indicate introns. c3’ss and c5’ss indicate the positions of activated intronic cryptic 3’ and 5’ splice sites, respectively, whereas n5’ss refers to the creation of a new 5’ss internal to exon 10. Dotted boxes represent pseudoexons (pEx), and numbers in brackets indicate the size of the RT-PCR products. (C) *In silico* predictions relative to the strength of the 5’splice sites (new 5’ss and reference 5’ss) described in (B). *In silico* analysis was performed for wild-type (WT), and *MLH1* variants c.840T>A and c.842C>T, by using three different algorithms (SSF, MES and HSF), as indicated. The coordinates of the last exonic position of each exon/intron junction defined by the aforementioned 5’ splice sites are indicated between brackets. SSF, SpliceSiteFinder-like; MES, MaxEntScan and HSF-ss, Human Splicing Finder- splice site dedicated.
(TIF)

S2 Fig Characterization of exon 10 skipping mutations by using a pCAS2-M1e10 minigene splicing reporter assay. (A) Structure of the minigenes used in the pCAS2-M1e10 splicing reporter assay. Boxes represent exons and lines in between indicate introns. The minigenes were generated by inserting a genomic fragment containing *MLH1* exon 10 and flanking intronic sequences (*MLH1* c.791-168_c.884+187) into the intron of pCAS2, as described under Materials and Methods. Arrows above the exons represent RT-PCR primers used in the splicing reporter assay. CMV, CMV promoter; Poly A, polyadenylation site. (B) Analysis of the splicing pattern of pCAS2-M1e10 minigenes. Wild-type (WT) and mutant pCAS2-M1e10 constructs were transfected into HeLa cells and then the minigenes’ transcripts were analyzed by RT-PCR as described under Materials and Methods. The top panel shows the RT-PCR products separated on a 2.5% agarose gel stained with ethidium bromide. The identities of the two major RT-PCR products, with or without exon 10 (a and b, respectively), are indicated on the left. The additional product c corresponds to exon 10 deleted of the last 48 nucleotides (splicing event explained in S1 Fig for product f). Product d is described in (C). The bottom panel shows the quantification of the RT-PCR products. Results are shown as the average of three independent experiments and are expressed as percentage of exon inclusion. Error bars indicate individual standard deviation (SD) values, whereas the horizontal dashed line delineates the lower limit of the SD bar observed for WT (93±2; i.e. 91% lower limit of exon inclusion). Variants producing exon inclusion levels under this value were considered as exon-skipping mutations. (C) Splicing events underlying the production of the RT-PCR product d visualized in (B). c5’ss indicates the position of an intronic cryptic 5’splice site activated in the presence of *MLH1* variants located at c.883 and c.884. The dotted box represents the resulting 150 nt- intronic retention. (D) *In silico* predictions relative to the strength of the 5’splice sites (cryptic 5’ss and reference 5’ss) described in (C). *In silico* analysis was performed by using three different algorithms (SSF, MES and HSF), as indicated. The coordinates of the last exonic position of each exon/intron junction defined by the aforementioned 5’ splice sites are indicated between brackets. SSF, SpliceSiteFinder-like; MES, MaxEntScan; and HSF-ss, Human Splicing Finder- splice site dedicated.
(TIF)

S3 Fig Splice site-dedicated bioinformatics tools can predict the impact on splicing of *MLH1* exon 10 variants. (A) Distribution of the seven *MLH1* exon 10 SNVs located within the reference 3’ and 5’ splice site consensus sequences (3’ss and 5’ss, respectively). The diagram shows the nucleotide composition of *MLH1* exon 10 (c.791-c.884), as well as the position and identity of each SNV. 3’ss and 5’ss indicate the position of reference exon/intron junctions. (B) Comparison of splice site-dedicated *in silico* predictions with experimental data obtained in the pSLP3m-M1e10 minigene splicing reporter assay. *In silico* analysis was performed for wild-type (WT) and the aforementioned mutants by using three different algorithms (SSF, MES and HSF-ss), as indicated. 3’ss and 5’ss refers to *MLH1* exon 10 reference 3’ and 5’ splice sites, respectively. SSF, SpliceSiteFinder-like; MES, MaxEntScan and HSF-ss, Human Splicing Finder- splice site dedicated; Δ, change relative to WT expressed as a percentage. The range of values is indicated into brackets for each tool. (C) Comparison of the 5’ss score change relative to WT obtained *in silico*, with exon 10 inclusion level change obtained in pSPL3m-M1e10 and pCAS2-M1e10 minigene assays for the last 6 mutations of exon 10.
(TIF)

S4 Fig Optimization of reaction conditions for semi-quantitative RT-PCR analysis of *MLH1* transcripts expressed in blood cells. (A) Preliminary RT-PCR reactions were performed with increasing number of PCR cycles in order to determine the linear range of the assay. The image shows the RT-PCR products obtained from fresh blood RNA of 3 healthy control individuals (C1, C2 and C3) and a patient carrying the heterozygous *MLH1* c.793C>T variant (P_793CT.1_), separated on a 2% agarose gel, as described under Materials and Methods. The identity of the RT-PCR products is indicated on the right of the gel. M, size marker (100 bp DNA ladder); FL, full-length; Δ, exon skipping. (B) Splicing events underlying the production of the RT-PCR products visualized in (A). Boxes represent exons and lines in between indicate introns. The different box shapes correspond to the phasing of each exon in terms of protein coding sequence, as illustrated in the lower right corner of the figure. Arrows above the exons symbolize the primers used in the RT-PCR reactions. The expected *MLH1* RNA (r.) and protein (p.) species are indicated on the right. Transcripts carrying a PTC are considered potential targets for nonsense-mediated decay (NMD). PTC, premature termination codon; FL, full-length; Δ, exon skipping.
(TIF)

S5 Fig Comparison of pCAS2-BRCA2 exon 7 minigene data [17] with results obtained from new ESR-dedicated bioinformatics tools. Top, middle and bottom panels refer to results obtained with ΔtESRseq-, ΔHZ_EI-_ and ΔΨ-based bioinformatics approaches, respectively, as described under Materials and Methods. *BRCA2* exon 7 variants located within the sequences that define the reference splice sites were eliminated from this analysis. Retained variants were separated into 2 groups depending on their impact on splicing as determined on the pCAS2-B2e7 minigene assay[17] and indicated above the graphs. P-values were calculated by using the Student’s t-test as described under Materials and Methods. Horizontal dashes delineate the upper thresholds.
(TIF)

S6 Fig Correlation analysis between *BRCA2* exon 7 inclusion levels [17] and results obtained from new ESR-dedicated bioinformatics tools. (A), (B) and (C) refer to results obtained with ΔtESRseq-, ΔHZ_EI-_ and ΔΨ-based bioinformatics approaches, respectively, as described under Materials and Methods. Only *BRCA2* exon 7 variants located outside the sequences that define the reference splice sites were retained for this analysis as already mentioned in S5 Fig. The precise correspondence between each Δ value (ΔtESRseq, ΔHZ_EI_ or ΔΨ), the level of exon inclusion observed in the pCAS2-B2e7 minigene assays [17], and the identity of the corresponding *BRCA2* exon 7 variant, is indicated on S2 Table Correlation coefficients (ρ) and p-values were determined by performing a Pearson correlation analysis, as described under Materials and Methods.
(TIF)

S7 Fig Detection of aberrant splicing in a patient carrying the heterozygous *MLH1* c.842C>T variant. (A) Relative position of *MLH1* SNVs detected in patients P_791-5TG.1_ (c.791-5T>G), P_842CT.1_ (c.842C>T) and P_882CT.1_ (c.882C>T). Boxes represent exons and lines in between indicate introns. The different box shapes correspond to the phasing of each exon in terms of protein coding sequence, as described in S4 Fig. Arrows above the exons represent primers used in RT-PCR reactions. (B) Comparative RT-PCR analysis of RNA samples obtained from puromycin-untreated and puromycin-treated LCLs (-Puro and +Puro, respectively) derived from 3 healthy control individuals (T401, T403 and T404) and patients carrying the heterozygous variants *MLH1* c.791-5T>G (P_791-5TG.1_), c.842C>T (P_842CT.1_) and c.882C>T (P_882CT.1_). RT-PCR reactions were performed with primers mapping to *MLH1* exon 8 (forward primer) and exon 12 (reverse primer), as described under Materials and Methods. The figure shows the RT-PCR products separated on a 2% agarose gel. RT-PCR product identifiers are indicated on the right of the gel. FL, full-length; Δ, exon skipping. (C) Assessment of *MLH1* allelic expression in patients P_791-5TG.1_ (c.791-5T>G), P_842CT.1_ (c.842C>T) and P_882CT.1_ (c.882C>T) by gDNA and cDNA sequencing, as indicated. Sequencing of gDNA and cDNA from T401, T403 and T404 (healthy controls described above) showed the absence of SNVs in *MLH1* exon 10 and the presence of homozygous SNP in exon 8 (T401 and T404, *MLH1* c.655AA; and T403, *MLH1* c.655GG). SNP Ex8, rs1799977 (*MLH1*c.655A>G, p.Ile219Val).
(TIF)

S1 Table Comparison of pSPL3m-M1e10 minigene data with bioinformatics predictions based on ΔtESRseq, ΔHZ_EI_ and ΔΨ values. (DOCX)

S2 Table Comparison of minigene splicing data with ESR-dedicated bioinformatics predictions for *MLH1* exon 10 variants. (DOC)

S3 Table Comparison of minigene splicing data with ESR-dedicated bioinformatics predictions for *BRCA2* exon 7 variants. (DOC)

S4 Table Comparison of minigene splicing data with ESR-dedicated bioinformatics predictions for *BRCA1* exon 6 variants. (DOC)

S5 Table Comparison of minigene splicing data with ESR-dedicated bioinformatics predictions for *CFTR* exon 12 variants. (DOC)

S6 Table Comparison of minigene splicing data with ESR-dedicated bioinformatics predictions for *NF1* exon 37 variants. (DOC)

S7 Table Comparative statistical analysis of the predictive power of four ESR-dedicated bioinformatics approaches by using five independent datasets. (DOC)

S8 Table Comparative analysis of the sensitivity and specificity of ESR-dedicated bioinformatics approaches in predicting exon-skipping mutations by using five independent datasets. (DOC)

S9 Table Explanations for the exceptions in the total number of variants taken into account in statistical analyses described in S3, S7 and S8 Tables. (DOC)
