# Correction: Exonic Splicing Mutations Are More Prevalent than Currently Estimated and Can Be Predicted by Using *In Silico* Tools

**DOI:** 10.1371/journal.pgen.1005971

**Published:** 2016-04-06

**Authors:** Omar Soukarieh, Pascaline Gaildrat, Mohamad Hamieh, Aurélie Drouet, Stéphanie Baert-Desurmont, Thierry Frébourg, Mario Tosi, Alexandra Martins

There is an error in Fig 4. The symbol for correlation coefficient "ρ" should be replaced with "r", to match the description in the main text and Figure legend. Please see the correct Fig 4 here.

**Fig 4:**
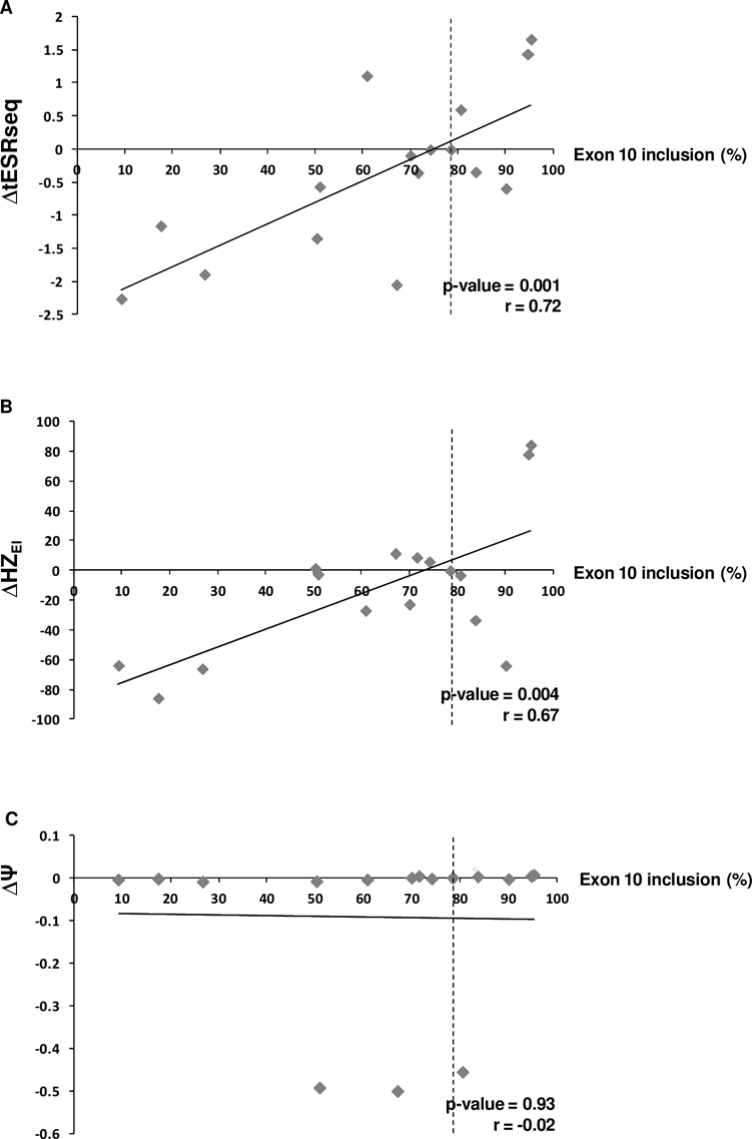
Correlation analysis between exon 10 inclusion levels and results obtained from new ESR-dedicated bioinformatics tools. (A), (B) and (C) refer to results obtained with ΔtESRseq-, ΔHZ_EI-_ and ΔΨ-based bioinformatics approaches, respectively, as described under Materials and Methods. Only *MLH1* exon 10 variants located outside the sequences that define the reference splice sites were retained for this analysis as already mentioned in Fig 3. The precise correspondence between each Δ value (ΔtESRseq, ΔHZ_EI_ or ΔΨ), the level of exon inclusion observed in the pSPL3m-M1e10 minigene assay, and the identity of the corresponding *MLH1* exon 10 variant, is indicated on S1 Table. Correlation coefficients (r) and p-values were determined by performing a Pearson correlation analysis, as described under Materials and Methods.

There is a typographical error in the last sentence of the paragraph following Fig 4 (Results section): ‘ΔΔHZEI’ should be replaced with ‘ΔHZEI’. The correct sentence is: “Moreover, given their lack of comprehensiveness and global quantitation, the performance of these two in silico tools could not be statistically analyzed, nor properly compared to that of ΔtESRseq, ΔHZEI, ΔΨ or EX-SKIP.”
