# Supplementary figures and images for: Exonic Splicing Mutations Are More Prevalent than Currently Estimated and Can Be Predicted by Using In Silico Tools
Source: PLoS Genet. 2016 Jan 13;12(1):e1005756. doi: 10.1371/journal.pgen.1005756 (PMC4711968; doi:10.1371/journal.pgen.1005756)

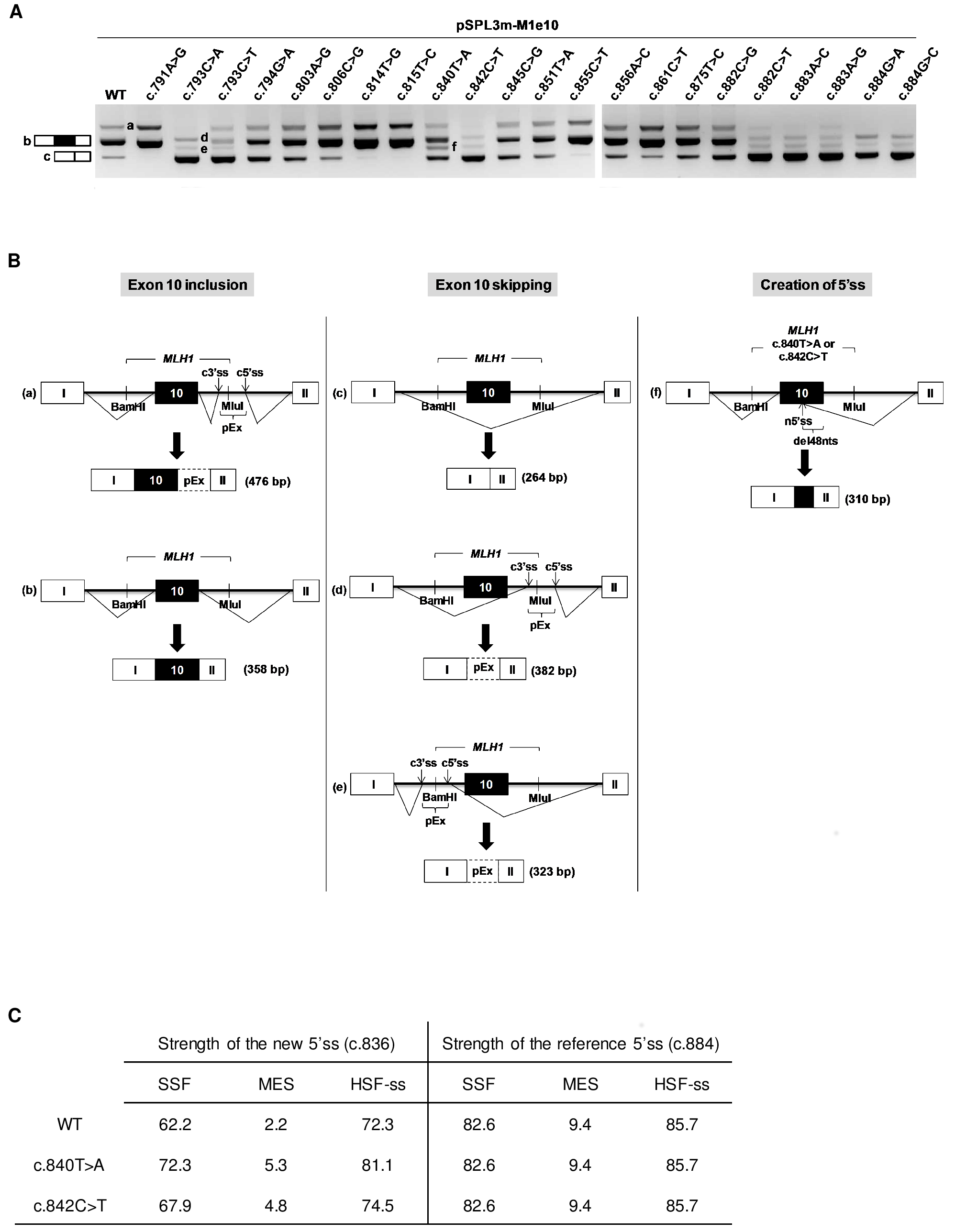

Supplement: S1 Fig — (A) Comparative RT-PCR analysis of the splicing patterns of wild-type and mutant pSPL3m-M1e10 minigenes, as indicated. The image shows the RT-PCR products separated on a 2.5% agarose gel as described in Fig 1C. (B) Splicing events underlying the production of the major (a, b and c) and minor (d, e and f) RT-PCR products visualized in (A). As indicated, RT-PCR products were separated into 3 groups according to the splicing behavior of MLH1 exon 10. Boxes represent exons and lines in between indicate introns. c3’ss and c5’ss indicate the positions of activated intronic cryptic 3’ and 5’ splice sites, respectively, whereas n5’ss refers to the creation of a new 5’ss internal to exon 10. Dotted boxes represent pseudoexons (pEx), and numbers in brackets indicate the size of the RT-PCR products. (C) In silico predictions relative to the strength of the 5’splice sites (new 5’ss and reference 5’ss) described in (B). In silico analysis was performed for wild-type (WT), and MLH1 variants c.840T>A and c.842C>T, by using three different algorithms (SSF, MES and HSF), as indicated. The coordinates of the last exonic position of each exon/intron junction defined by the aforementioned 5’ splice sites are indicated between brackets. SSF, SpliceSiteFinder-like; MES, MaxEntScan and HSF-ss, Human Splicing Finder- splice site dedicated. (TIF) [file pgen.1005756.s001.tif]

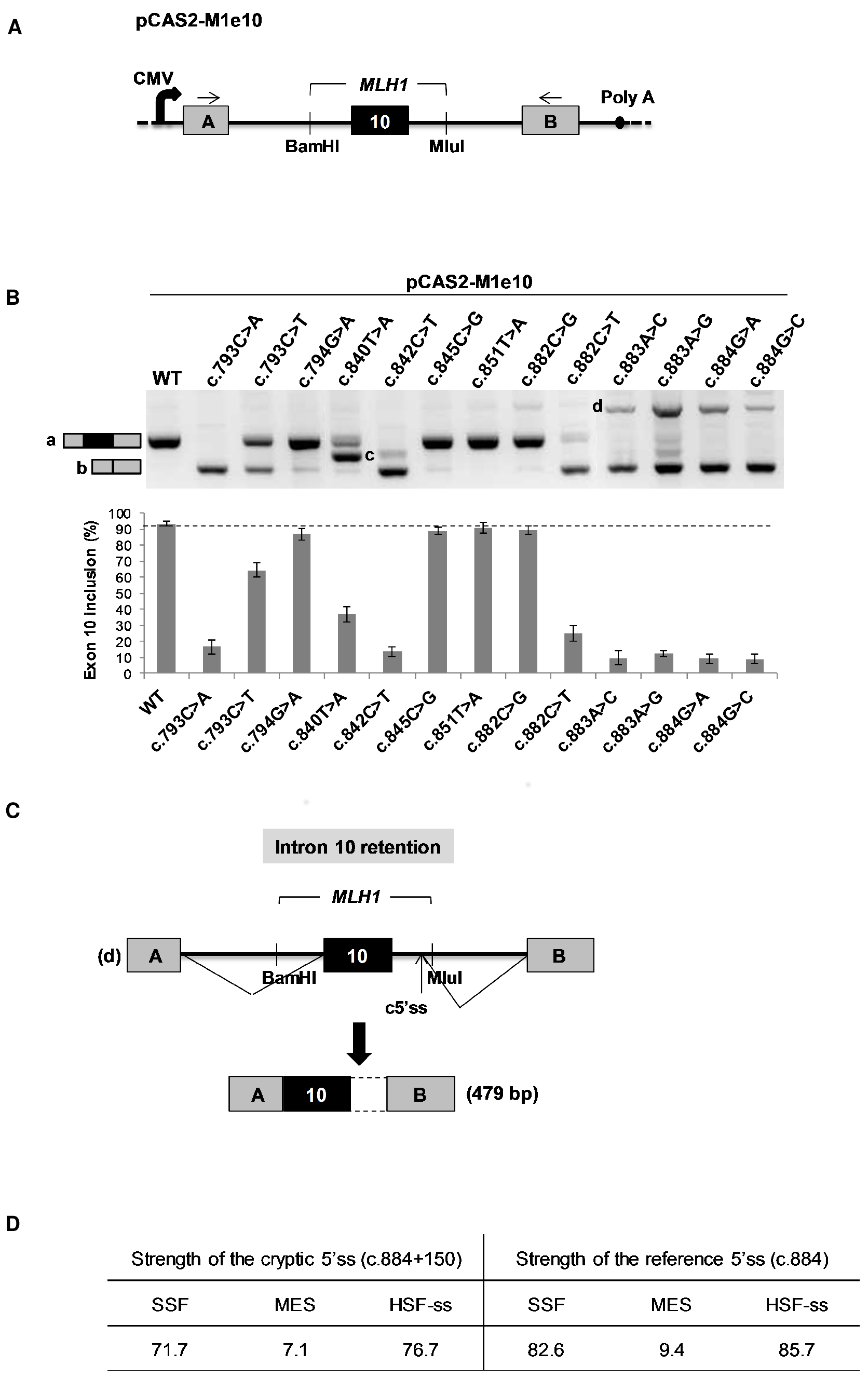

Supplement: S2 Fig — (A) Structure of the minigenes used in the pCAS2-M1e10 splicing reporter assay. Boxes represent exons and lines in between indicate introns. The minigenes were generated by inserting a genomic fragment containing MLH1 exon 10 and flanking intronic sequences (MLH1 c.791-168_c.884+187) into the intron of pCAS2, as described under Materials and Methods. Arrows above the exons represent RT-PCR primers used in the splicing reporter assay. CMV, CMV promoter; Poly A, polyadenylation site. (B) Analysis of the splicing pattern of pCAS2-M1e10 minigenes. Wild-type (WT) and mutant pCAS2-M1e10 constructs were transfected into HeLa cells and then the minigenes’ transcripts were analyzed by RT-PCR as described under Materials and Methods. The top panel shows the RT-PCR products separated on a 2.5% agarose gel stained with ethidium bromide. The identities of the two major RT-PCR products, with or without exon 10 (a and b, respectively), are indicated on the left. The additional product c corresponds to exon 10 deleted of the last 48 nucleotides (splicing event explained in S1 Fig for product f). Product d is described in (C). The bottom panel shows the quantification of the RT-PCR products. Results are shown as the average of three independent experiments and are expressed as percentage of exon inclusion. Error bars indicate individual standard deviation (SD) values, whereas the horizontal dashed line delineates the lower limit of the SD bar observed for WT (93±2; i.e. 91% lower limit of exon inclusion). Variants producing exon inclusion levels under this value were considered as exon-skipping mutations. (C) Splicing events underlying the production of the RT-PCR product d visualized in (B). c5’ss indicates the position of an intronic cryptic 5’splice site activated in the presence of MLH1 variants located at c.883 and c.884. The dotted box represents the resulting 150 nt- intronic retention. (D) In silico predictions relative to the strength of the 5’splice sites (cryptic 5’ss and [file pgen.1005756.s002.tif]

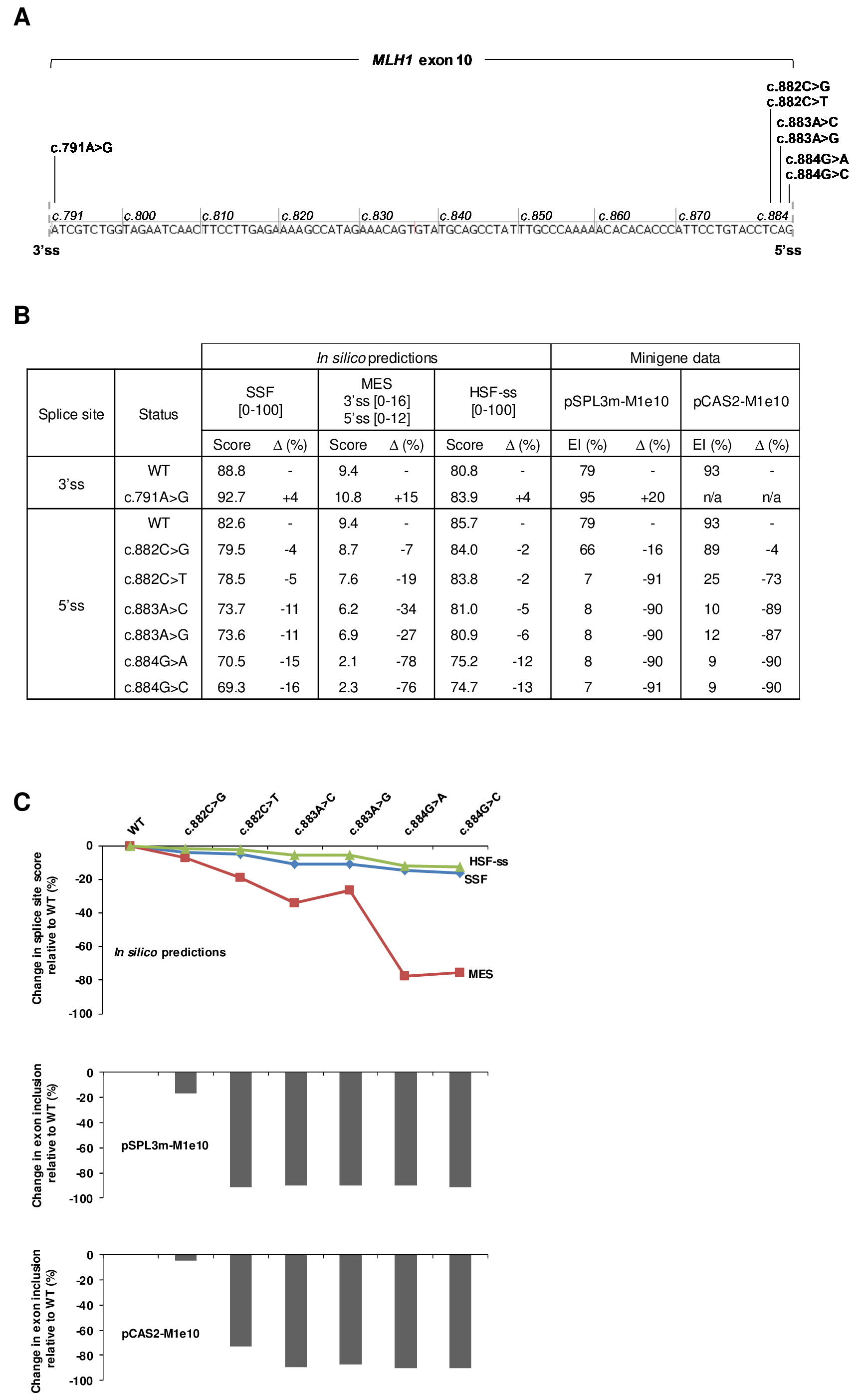

Supplement: S3 Fig — (A) Distribution of the seven MLH1 exon 10 SNVs located within the reference 3’ and 5’ splice site consensus sequences (3’ss and 5’ss, respectively). The diagram shows the nucleotide composition of MLH1 exon 10 (c.791-c.884), as well as the position and identity of each SNV. 3’ss and 5’ss indicate the position of reference exon/intron junctions. (B) Comparison of splice site-dedicated in silico predictions with experimental data obtained in the pSLP3m-M1e10 minigene splicing reporter assay. In silico analysis was performed for wild-type (WT) and the aforementioned mutants by using three different algorithms (SSF, MES and HSF-ss), as indicated. 3’ss and 5’ss refers to MLH1 exon 10 reference 3’ and 5’ splice sites, respectively. SSF, SpliceSiteFinder-like; MES, MaxEntScan and HSF-ss, Human Splicing Finder- splice site dedicated; Δ, change relative to WT expressed as a percentage. The range of values is indicated into brackets for each tool. (C) Comparison of the 5’ss score change relative to WT obtained in silico, with exon 10 inclusion level change obtained in pSPL3m-M1e10 and pCAS2-M1e10 minigene assays for the last 6 mutations of exon 10. (TIF) [file pgen.1005756.s003.tif]

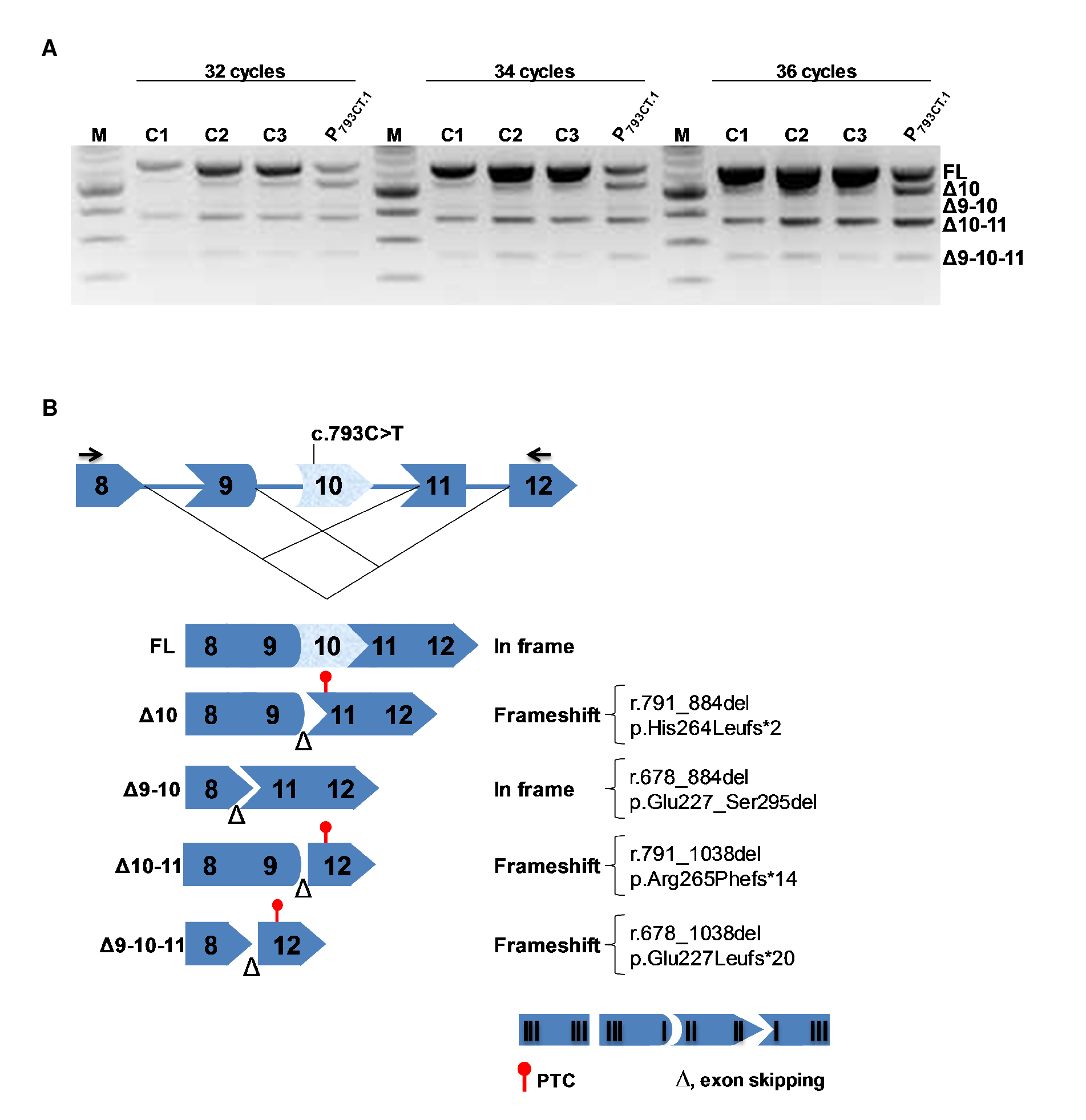

Supplement: S4 Fig — (A) Preliminary RT-PCR reactions were performed with increasing number of PCR cycles in order to determine the linear range of the assay. The image shows the RT-PCR products obtained from fresh blood RNA of 3 healthy control individuals (C1, C2 and C3) and a patient carrying the heterozygous MLH1 c.793C>T variant (P793CT.1), separated on a 2% agarose gel, as described under Materials and Methods. The identity of the RT-PCR products is indicated on the right of the gel. M, size marker (100 bp DNA ladder); FL, full-length; Δ, exon skipping. (B) Splicing events underlying the production of the RT-PCR products visualized in (A). Boxes represent exons and lines in between indicate introns. The different box shapes correspond to the phasing of each exon in terms of protein coding sequence, as illustrated in the lower right corner of the figure. Arrows above the exons symbolize the primers used in the RT-PCR reactions. The expected MLH1 RNA (r.) and protein (p.) species are indicated on the right. Transcripts carrying a PTC are considered potential targets for nonsense-mediated decay (NMD). PTC, premature termination codon; FL, full-length; Δ, exon skipping. (TIF) [file pgen.1005756.s004.tif]

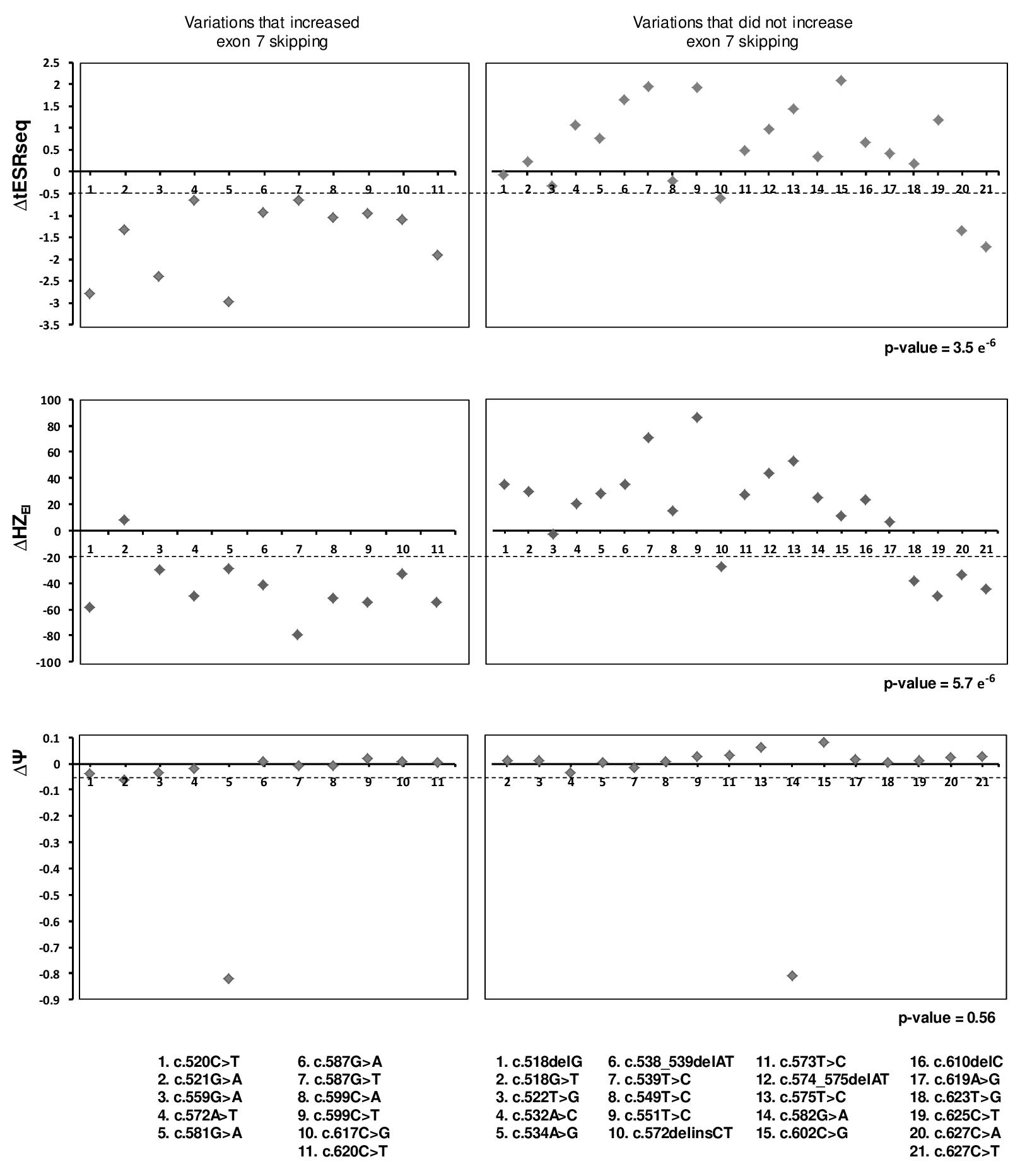

Supplement: S5 Fig — Top, middle and bottom panels refer to results obtained with ΔtESRseq-, ΔHZEI- and ΔΨ-based bioinformatics approaches, respectively, as described under Materials and Methods. BRCA2 exon 7 variants located within the sequences that define the reference splice sites were eliminated from this analysis. Retained variants were separated into 2 groups depending on their impact on splicing as determined on the pCAS2-B2e7 minigene assay[17] and indicated above the graphs. P-values were calculated by using the Student’s t-test as described under Materials and Methods. Horizontal dashes delineate the upper thresholds. (TIF) [file pgen.1005756.s005.tif]

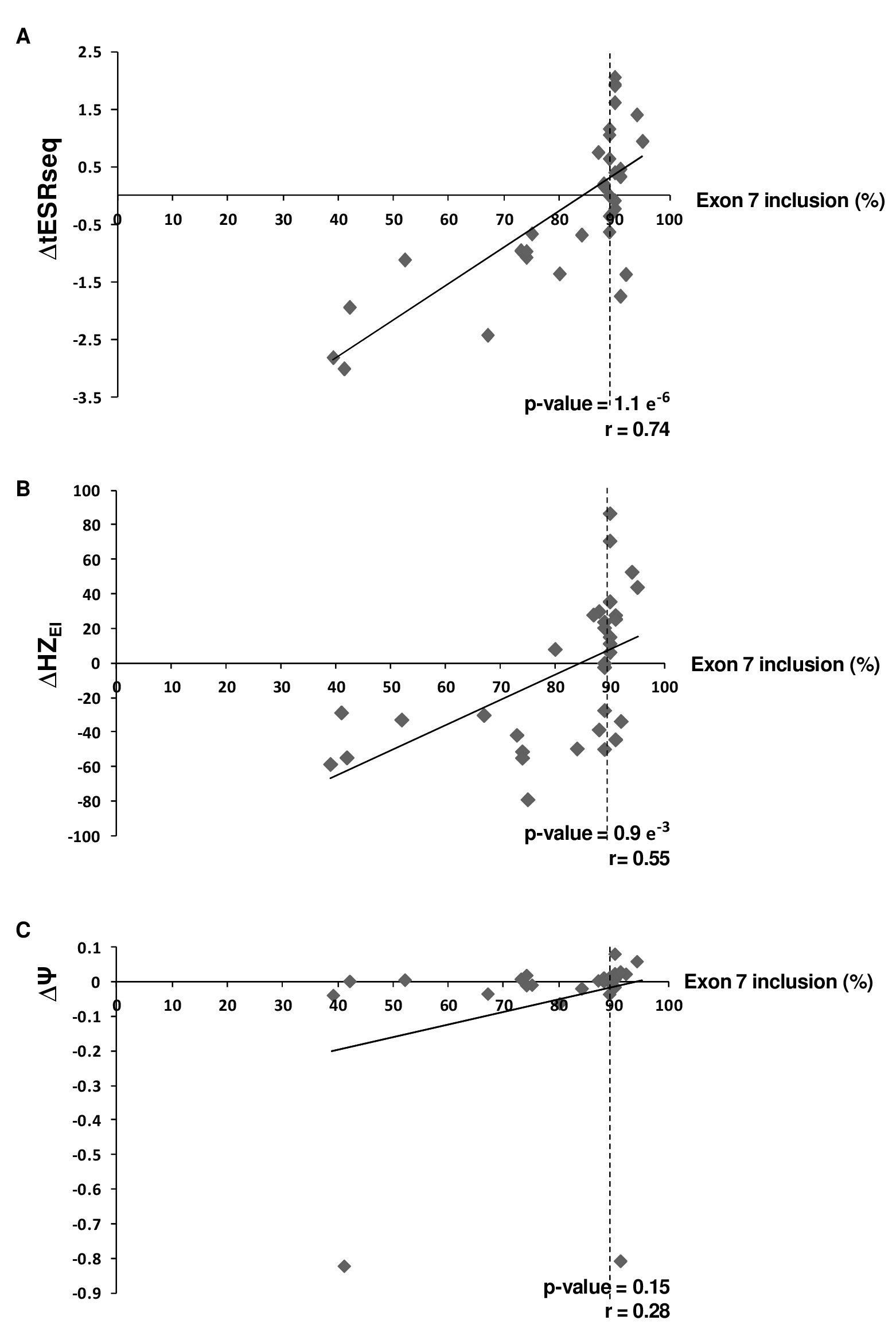

Supplement: S6 Fig — (A), (B) and (C) refer to results obtained with ΔtESRseq-, ΔHZEI- and ΔΨ-based bioinformatics approaches, respectively, as described under Materials and Methods. Only BRCA2 exon 7 variants located outside the sequences that define the reference splice sites were retained for this analysis as already mentioned in S5 Fig. The precise correspondence between each Δ value (ΔtESRseq, ΔHZEI or ΔΨ), the level of exon inclusion observed in the pCAS2-B2e7 minigene assays [17], and the identity of the corresponding BRCA2 exon 7 variant, is indicated on S2 Table Correlation coefficients (ρ) and p-values were determined by performing a Pearson correlation analysis, as described under Materials and Methods. (TIF) [file pgen.1005756.s006.tif]

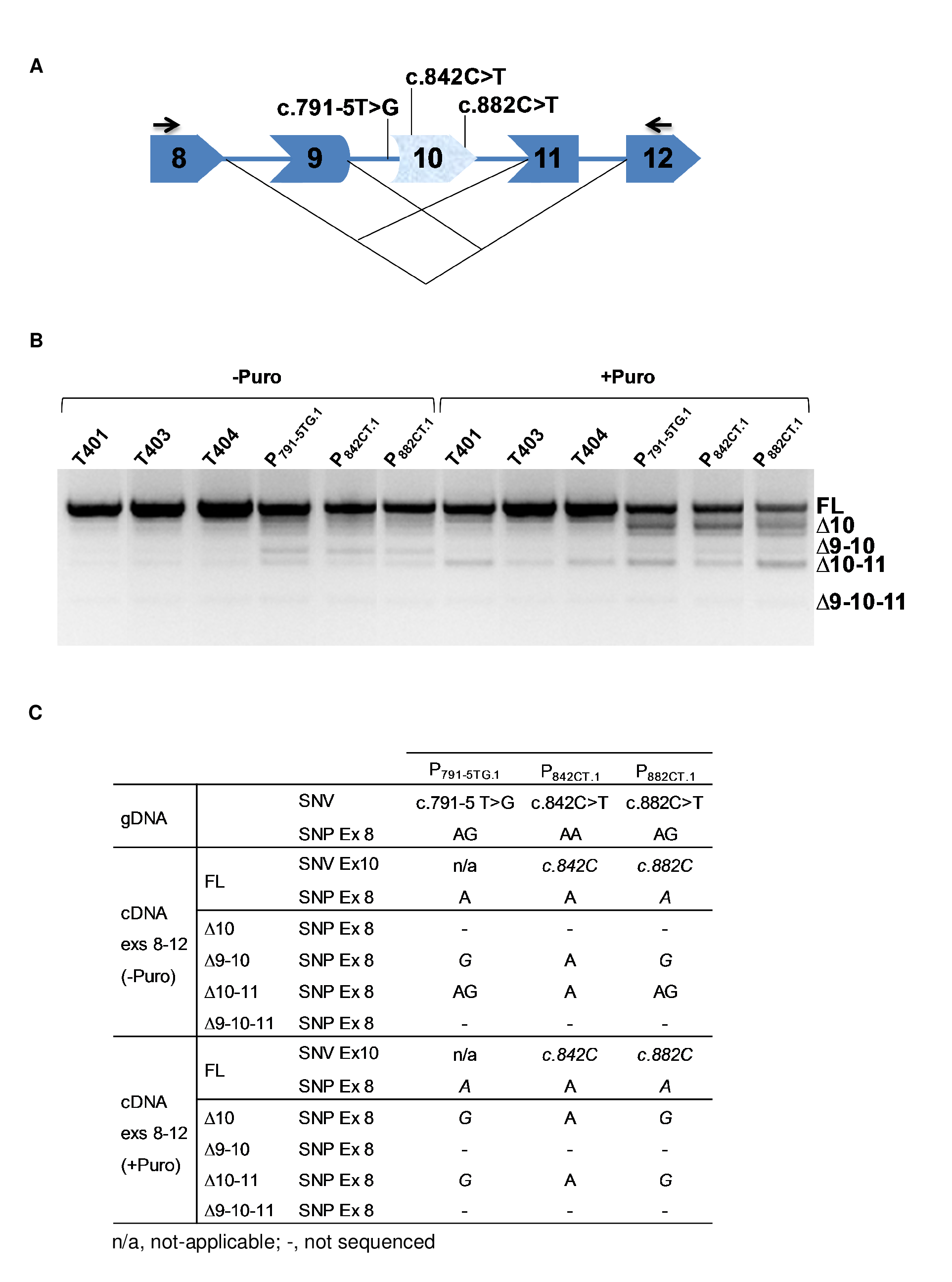

Supplement: S7 Fig — (A) Relative position of MLH1 SNVs detected in patients P791-5TG.1 (c.791-5T>G), P842CT.1 (c.842C>T) and P882CT.1 (c.882C>T). Boxes represent exons and lines in between indicate introns. The different box shapes correspond to the phasing of each exon in terms of protein coding sequence, as described in S4 Fig. Arrows above the exons represent primers used in RT-PCR reactions. (B) Comparative RT-PCR analysis of RNA samples obtained from puromycin-untreated and puromycin-treated LCLs (-Puro and +Puro, respectively) derived from 3 healthy control individuals (T401, T403 and T404) and patients carrying the heterozygous variants MLH1 c.791-5T>G (P791-5TG.1), c.842C>T (P842CT.1) and c.882C>T (P882CT.1). RT-PCR reactions were performed with primers mapping to MLH1 exon 8 (forward primer) and exon 12 (reverse primer), as described under Materials and Methods. The figure shows the RT-PCR products separated on a 2% agarose gel. RT-PCR product identifiers are indicated on the right of the gel. FL, full-length; Δ, exon skipping. (C) Assessment of MLH1 allelic expression in patients P791-5TG.1 (c.791-5T>G), P842CT.1 (c.842C>T) and P882CT.1 (c.882C>T) by gDNA and cDNA sequencing, as indicated. Sequencing of gDNA and cDNA from T401, T403 and T404 (healthy controls described above) showed the absence of SNVs in MLH1 exon 10 and the presence of homozygous SNP in exon 8 (T401 and T404, MLH1 c.655AA; and T403, MLH1 c.655GG). SNP Ex8, rs1799977 (MLH1c.655A>G, p.Ile219Val). (TIF) [file pgen.1005756.s007.tif]
