# Supplementary material for: Exonic Splicing Mutations Are More Prevalent than Currently Estimated and Can Be Predicted by Using In Silico Tools
Source: PLoS Genet. 2016 Jan 13;12(1):e1005756. doi: 10.1371/journal.pgen.1005756 (PMC4711968; doi:10.1371/journal.pgen.1005756)
Supplement: S1 Table — (DOCX) [file pgen.1005756.s008.docx]

**S1 Table. Comparison of pSPL3m-M1e10 minigene splicing data with bioinformatics predictions based on ∆tESRseq, ∆HZ_EI_ and ∆Ψ** [1] **values.** The table shows 15 *MLH1* variants located outside the reference splice sites (7 variants that increased exon 10 skipping, 5 variants with no effect on splicing and 3 that increased exon inclusion). True and false calls (color code indicated underneath the table) were determined based on variant-induced exon skipping events and taking into account the following upper thresholds: -0.5 for ∆tESRseq, -20 for ∆HZ_EI_, and -0.05 for ∆Ψ, as described under Material and Methods.

| *MLH1* variant  (n=15) | Effect on splicing | Exon 10 inclusion (%) | Increased exon skipping | ∆tESRseq | ∆HZ_EI_ | ∆Ψ |
| --- | --- | --- | --- | --- | --- | --- |
| WT | n/a | 79 | n/a | 0 | 0 | 0 |
| c.793C>A | Increased exon skipping | 9 | Yes | -2.25 | -63.57 | -0.004 |
| c.793C>T | Increased exon skipping | 27 | Yes | -1.88 | -65.88 | -0.009 |
| c.794G>A | Increased exon skipping | 50 | Yes | -1.34 | 1.56 | -0.008 |
| c.840T>A | Increased exon skipping | 51 | Yes | -0.56 | -2.39 | -0.493 |
| c.842C>T | Increased exon skipping | 18 | Yes | -1.15 | -85.57 | -0.002 |
| c.845C>G | Increased exon skipping | 61 | Yes | 1.11 | -26.75 | -0.004 |
| c.851T>A | Increased exon skipping | 67 | Yes | -2.04 | 11.5 | -0.501 |
| c.803A>G | No effect | 74 | No | -0.002 | 5.95 | -0.002 |
| c.806C>G | No effect | 81 | No | 0.59 | -3.09 | -0.456 |
| c.856A>C | No effect | 70 | No | -0.09 | -22.63 | 0 |
| c.861C>T | No effect | 84 | No | -0.34 | -33.27 | 0.003 |
| c.875T>C | No effect | 72 | No | -0.35 | 8.83 | 0.004 |
| c.814T>G | Increased exon inclusion | 95 | No | 1.42 | 78.03 | 0.004 |
| c.815T>C | Increased exon inclusion | 95 | No | 1.66 | 84.35 | 0.007 |
| c.855C>T | Increased exon inclusion | 90 | No | -0.59 | -63.76 | -0.003 |
| **True**  **calls** | | | Positive | 6 | 4 | 2 |
|  |  |  | Negative | 7 | 5 | 7 |
|  |  |  | **Total** | **13** | **9** | **9** |
| **False**  **calls** | | | Positive | 1 | 3 | 1 |
|  |  |  | Negative | 1 | 3 | 5 |
|  |  |  | **Total** | **2** | **6** | **6** |
| Sensitivity (%) | | | | 86 | 57 | 29 |
| Specificity (%) | | | | 88 | 63 | 88 |

| **True positive calls** | **True negative calls** | **False positive calls** | **False negative calls** |
| --- | --- | --- | --- |

1. Xiong HY, Alipanahi B, Lee LJ, Bretschneider H, Merico D, Yuen RKC, et al. The human splicing code reveals new insights into the genetic determinants of disease. Science. 2014; doi:10.1126/science.1254806
