# Supplementary material for: Exonic Splicing Mutations Are More Prevalent than Currently Estimated and Can Be Predicted by Using In Silico Tools
Source: PLoS Genet. 2016 Jan 13;12(1):e1005756. doi: 10.1371/journal.pgen.1005756 (PMC4711968; doi:10.1371/journal.pgen.1005756)
Supplement: S2 Table — (DOC) [file pgen.1005756.s009.doc]

**S2 Table. Comparison of minigene splicing data with ESR-dedicated bioinformatics predictions for *MLH1* exon 10 variants.**  The impact on splicing of 15 *MLH1* exon 10 variants located outside the reference splice sites was determined in the context of the pSPL3m-MLH1-exon 10 minigene, as described under Materials and Methods. The table shows a separation of the variants into 2 groups according to the pSPL3m-M1e10 minigene results: variants that increased exon skipping (n=7) and those that did not (n=8, including 5 variants with no effect and 3 that increased exon inclusion, as indicated by the separating line). *In silico* predictions of potential effects on splicing were conducted by using 3 newly developed ESR-dedicated approaches (ΔtESRseq, ΔHZ_EI_ and ΔΨ [1]), as well as 3 prior methods (EX-SKIP, ESEfinder and HSF-SR), as described under Materials and Methods. True and false calls (color code indicated underneath the table) of exon-skipping events were determined by taking into account the following thresholds: -0.5 for ∆tESRseq, -20 for ∆HZ_EI_, -0.05 for ∆Ψ and 1 for EX-SKIP. n/a, not applicable; nd, not determined due to the presence of conflicting calls (e.g. simultaneous indication of destruction/weakening and creation/strengthening of ESEs for a given variant). *, ESEfinder and HSF-SR scores of potential ESEs (SC35, SF2/ASF, SF2/ASF (IgM-BRCA1), SRp40, SRp55 and Tra2) and ESS (hnRNPA1) are indicated in the WT and variant contexts (WT►variant). ^a^, ESEfinder; ^b^, exon-identity element (EIE) and intron-identity element (IIE) [2] ; ^c^, [3] ; ^d^, experimental ; ^e^, Rescue ESE ; ^f^, 8-mers from [4]; ^g^, Fas-ESS hexamers.

|  | *MLH1* variant  (n=15) | New *In silico* approaches | | | Prior *in silico* approaches | | | |
| --- | --- | --- | --- | --- | --- | --- | --- | --- |
|  |  | ∆tESRseq | ∆HZ_EI_ | ∆Ψ | EX-SKIP  (ESE/ESS) | ESEfinder* | HSF-SR* | |
|  |  |  |  |  |  |  | ESE | ESS |
|  | WT | 0 | 0 | 0 | 1 | n/a | n/a | n/a |
| Variants that increased  exon skipping  (n=7) | c.793C>A | -2.25 | -63.57 | -0.004 | 1 | SRp40: 3.85►3.16 | SRp40: 85.15►81.02 ^a^  EIE broken ^b^ | ESS: -►61.28 ^c^  hnRNP A1: 75►76.9;  -►73.81 ^d^ |
|  | c.793C>T | -1.88 | -65.88 | -0.009 | 0.90 | SRp40: 3.85►4.21 | SRp40: 85.15►87.31 ^a^ | ESS: -►68.11 ^c^  6 New IIEs ^b^  hnRNP A1: 75►75.95 ^d^ |
|  | c.794G>A | -1.34 | 1.56 | -0.008 | 1.04 | SC35: 3.31►-  SRp40: 3.85►- | SRp40: 85.15►- ^a^  SC35: 80.76►- ^a^  Tra2: -►60.94 ^d^  new ESE ^e^ | IIE broken ^b^ |
|  | c.840T>A | -0.56 | -2.39 | -0.493 | 1.13 | SC35: 2.64►-  SRp55: 4.72►2.84 | SC35: 76.64►- ^a^  SRp55: 86.98►74.95 ^a^  2 EIEs broken ^b^  new ESE ^e^  ESE: -►40.31 ^f^ | IIE broken ^b^  hnRNP A1: 70.72►72.86 ^d^  ESS: 27.69►- ^f^ |
|  | c.842C>T | -1.15 | -85.57 | -0.002 | 0.80 | SF2/ASF: 3.23►-  SF2/ASF (IgM-BRCA1): 3.07►-  SC35: 2.64►-  SRp55: -►3.61; 4.72►3.17 | SF2/ASF: 80.43►- ^a^  SF2/ASF (IgM-BRCA1): 79.77►-^a^  SC35: 76.64►- ^a^  SRp55: -►79.88; 86.98►77.06 ^a^  3 EIEs broken ^b^ | ESS: 27.69►- ^f^  4 new IIEs ^b^  hnRNP A1: -►73.81; 70.72►69.05 ^d^ |
|  | c.845C>G | 1.11 | -26.75 | -0.004 | 0.91 | SF2/ASF: 3.23►-  SF2/ASF (IgM-BRCA1): 3.07►-  SRp55: 4.72►2.95 | SF2/ASF: 80.43►- ^a^  SF2/ASF (IgM-BRCA1): 79.77►-^a^  SRp55: 86.98►75.65a  EIE broken ^b^  Tra2: 63.42►66.85 ^d^ | ESS: -►62.45 ^c^  hnRNP A1: -►73.81 ^d^  ESS: -►30.97 ^f^ |
|  | c.851T>A | -2.04 | 11.5 | -0.501 | 1.13 | no change | new EIE ^b^ | 3 IIEs broken ^b^  ESS: 64.4►- ; 77.37►76.49 ^c^  hnRNP A1: -►72.86 ^d^ |
| Variants that did not increase  exon skipping  (n=8) | c.803A>G | -0.002 | 5.95 | -0.002 | 0.96 | no change | Tra2: 70.74►-; -►60.34;  -/61.95 ^d^ | 2 new IIEs ^b^  ESSs: -►62.75; -►71.10 ^c^  hnRNP A1: 79.29►90.72 ^d^  new ESS ^g^ |
|  | c.806C>G | 0.59 | -3.09 | -0.456 | 1 | no change | 2 new EIEs ^b^  1 EIE broken ^b^  Tra2: -►61.95 ^d^  new ESE ^e^  ESE broken ^e^ | no change |
|  | c.856A>C | -0.09 | -22.63 | 0 | 1.04 | SC35: -►2.92  SRp40: -►2.98 | SC35: -►78.37 ^a^  SRp40: -►79.94 ^a^  1 new ESE ^e^  2 new EIEs ^b^  EIE broken ^b^  Tra2: 94.14►60.13 ^d^ | ESSs: -►75.6; -►65.35; 64.4►61.11; 77.37►66.26 ^c^ |
|  | c.861C>T | -0.34 | -33.27 | 0.003 | 1.04 | SF2/ASF (IgM-BRCA1): 2.21►- | SF2/ASF (IgM-BRCA1): 73.15►-^a^  3 new EIEs ^b^  Tra2: 81.02►85.42 ^d^ | ESS: 60.79►- ^c^  ESSs: 35.86►-; 41.21►30.43 ^f^ |
|  | c.875T>C | -0.35 | 8.83 | 0.004 | 1.10 | SC35: 3.25►2.61 | SC35: 80.39►76.46 ^a^  EIE broken ^b^ | 2 IIEs broken ^b^  ESSs: -►63.53; 61.04►- ^c^  ESS broken ^g^ |
|  | c.814T>G | 1.42 | 78.03 | 0.004 | 1.13 | SF2/ASF (IgM-BRCA1):  -►2.27  SC35: -►2.7 | SC35: -►77.01 ^a^  SF2/ASF (IgM-BRCA1):  -►73.62 ^a^  ESE: 34.29►- ^f^ | 5 broken IIEs ^b^  ESSs: 63.11►-; 63.18►62.55 ^c^ |
|  | c.815T>C | 1.66 | 84.35 | 0.007 | 1.23 | SRp40: -►3.06 | SRp40: -►80.42 ^a^  new EIE ^b^  ESE broken ^e^  ESEs: -►46.74; 34.29►32.28 ^f^ | 4 new IIEs ^b^  ESS: 63.11►66.71 ^c^  1 ESS broken ^g^ |
|  | c.855C>T | -0.59 | -63.76 | -0.003 | 0.98 | SRp55: -►3.47 | SRp55: -►78.98 ^a^  3 new EIEs ^b^ | new IIE ^b^  ESSs: 64.4►66.1; 77.37►60.19 ^c^ |
| **True**  **calls** | Positive | 6 | 4 | 2 | 3 | 4 | 1 | 3 |
|  | Negative | 7 | 5 | 7 | 6 | 6 | 1 | 3 |
|  | **Total** | **13** | **9** | **9** | **9** | **10** | **2** | **6** |
| **False**  **calls** | Positive | 1 | 3 | 1 | 2 | 2 | 1 | 1 |
|  | Negative | 1 | 3 | 5 | 4 | 2 | 2 | 1 |
|  | **Total** | **2** | **6** | **6** | **6** | **4** | **3** | **2** |
| Conflicting calls | | 0 | 0 | 0 | 0 | 1 | 10 | 7 |
| Sensitivity (%) | | 86 | 57 | 29 | 43 | nd | nd | nd |
| Specificity (%) | | 88 | 63 | 88 | 75 | nd | nd | nd |

| **True positive calls** | **True negative calls** | **False positive calls** | **False negative calls** | **Conflicting calls** |
| --- | --- | --- | --- | --- |

1. Xiong HY, Alipanahi B, Lee LJ, Bretschneider H, Merico D, Yuen RKC, et al. The human splicing code reveals new insights into the genetic determinants of disease. Science. 2014; doi:10.1126/science.1254806

2. Zhang C, Li W-H, Krainer AR, Zhang MQ. RNA landscape of evolution for optimal exon and intron discrimination. Proc Natl Acad Sci U S A. 2008;105: 5797–5802. doi:10.1073/pnas.0801692105

3. Sironi M, Menozzi G, Riva L, Cagliani R, Comi GP, Bresolin N, et al. Silencer elements as possible inhibitors of pseudoexon splicing. Nucleic Acids Res. 2004;32: 1783–1791. doi:10.1093/nar/gkh341

4. Zhang XH-F, Chasin LA. Computational definition of sequence motifs governing constitutive exon splicing. Genes Dev. 2004;18: 1241–1250. doi:10.1101/gad.1195304
