# Supplementary material for: Exonic Splicing Mutations Are More Prevalent than Currently Estimated and Can Be Predicted by Using In Silico Tools
Source: PLoS Genet. 2016 Jan 13;12(1):e1005756. doi: 10.1371/journal.pgen.1005756 (PMC4711968; doi:10.1371/journal.pgen.1005756)
Supplement: S3 Table — (DOC) [file pgen.1005756.s010.doc]

**S3 Table. Comparison of minigene splicing data with ESR-dedicated bioinformatics predictions for *BRCA2* exon 7 variants.** The effect on splicing of 32 *BRCA2* exon 7 variants located outside the reference splice sites was previously determined in a pCAS2-BRCA2-exon 7 minigene assay [1]. The table shows a separation of the variants into 2 groups according to the minigene results: variants that increased exon skipping (n=11) and those that did not (n=21 variants). *In silico* predictions of potential effects on splicing were conducted by using 3 newly developed on ESR-dedicated approaches (ΔtESRseq, ΔHZ_EI_ and ΔΨ), as well as by using one prior method, (EX-SKIP), as described under Materials and Methods. True and false calls (color codes indicated underneath the table) of exon-skipping events were determined by taking into account the following thresholds: -0.5 for ∆tESRseq, -20 for ∆HZ_EI_, -0.05 for ∆Ψ, and 1 for EX-SKIP. n/a, not applicable; *, the total number of variants taken into consideration in the ∆Ψ analysis was 27 instead of 32, because ∆Ψ values cannot be calculated for del/ins variants.

|  | *BRCA2*variant  (n=32) | Exon 7 inclusion  (%) | New *in silico* methods | | | Prior *in silico* method |
| --- | --- | --- | --- | --- | --- | --- |
|  |  |  | ∆tESRseq | ∆HZ_EI_ | ∆Ψ | EX-SKIP  (ESE/ESS) |
|  | WT | 89 | 0 | 0 | 0 | 1 |
| Variants that increased  exon skipping  (n=11) | c.520C>T | 39 | -2.81 | -58.56 | -0.037 | 0.91 |
|  | c.521G>A | 80 | -1.35 | 7.97 | -0.061 | 1.06 |
|  | c.559G>A | 67 | -2.42 | -30.18 | -0.033 | 0.93 |
|  | c.572A>T | 84 | -0.68 | -49.55 | -0.018 | 0.88 |
|  | c.581G>A | 41 | -3.006 | -28.74 | -0.821 | 0.97 |
|  | c.587G>A | 73 | -0.95 | -41.70 | 0.009 | 1.01 |
|  | c.587G>T | 75 | -0.66 | -79.01 | -0.007 | 0.97 |
|  | c.599C>A | 74 | -1.07 | -51.25 | -0.008 | 1.04 |
|  | c.599C>T | 74 | -0.96 | -54.91 | 0.02 | 1 |
|  | c.617C>G | 52 | -1.11 | -32.80 | 0.006 | 0.94 |
|  | c.620C>T | 42 | -1.93 | -54.72 | 0.003 | 0.88 |
| Variants that did not increase  exon skipping  (n=21) | c.518delG | 90 | -0.08 | 35.53 | n/a | 1 |
|  | c.518G>T | 88 | 0.204 | 29.88 | 0.013 | 0.93 |
|  | c.522T>G | 89 | -0.35 | -2.44 | 0.013 | 0.97 |
|  | c.532A>C | 89 | 1.05 | 20.35 | -0.034 | 0.96 |
|  | c.534A>G | 87 | 0.74 | 27.87 | 0.004 | 1.01 |
|  | c.538_539delAT | 90 | 1.61 | 35.5 | n/a | 1.10 |
|  | c.539T>C | 90 | 1.93 | 70.66 | -0.013 | 1.11 |
|  | c.549T>C | 90 | -0.22 | 15.08 | 0.009 | 1.12 |
|  | c.551T>C | 90 | 1.91 | 86.59 | 0.025 | 1.10 |
|  | c.572delinsCT | 89 | -0.63 | -27.37 | n/a | 1 |
|  | c.573T>C | 91 | 0.46 | 27.67 | 0.0307 | 1.12 |
|  | c.574_575delAT | 95 | 0.94 | 43.91 | n/a | 1.10 |
|  | c.575T>C | 94 | 1.403 | 52.7 | 0.0605 | 1.23 |
|  | c.582G>A | 91 | 0.33 | 25.42 | -0.806 | 1.06 |
|  | c.602C>G | 90 | 2.05 | 11.29 | 0.081 | 1.03 |
|  | c.610delC | 89 | 0.63 | 23.76 | n/a | 0.96 |
|  | c.619A>G | 90 | 0.39 | 6.23 | 0.016 | 0.99 |
|  | c.623T>G | 88 | 0.16 | -38.56 | 0.002 | 0.99 |
|  | c.625C>T | 89 | 1.15 | -49.85 | 0.013 | 0.84 |
|  | c.627C>A | 92 | -1.36 | -33.69 | 0.024 | 1.03 |
|  | c.627C>T | 91 | -1.74 | -44.26 | 0.027 | 0.93 |
| **True calls** | | Positive | 11 | 10 | 2 | 7 |
|  |  | Negative | 18 | 16 | 15 | 13 |
|  |  | **Total** | **29** | **26** | **17*** | **20** |
| **False calls** | | Positive | 3 | 5 | 1 | 8 |
|  |  | Negative | 0 | 1 | 9 | 4 |
|  |  | **Total** | **3** | **6** | **10*** | **12** |
| Sensitivity (%) | | | 100 | 91 | 18***** | 64 |
| Specificity (%) | | | 86 | 76 | 94***** | 62 |

| **True positive calls** | **True negative calls** | **False positive calls** | **False negative calls** |
| --- | --- | --- | --- |

1. Di Giacomo D, Gaildrat P, Abuli A, Abdat J, Frébourg T, Tosi M, et al. Functional analysis of a large set of BRCA2 exon 7 variants highlights the predictive value of hexamer scores in detecting alterations of exonic splicing regulatory elements. Hum Mutat. 2013;34: 1547–1557. doi:10.1002/humu.22428
