# Supplementary material for: Exonic Splicing Mutations Are More Prevalent than Currently Estimated and Can Be Predicted by Using In Silico Tools
Source: PLoS Genet. 2016 Jan 13;12(1):e1005756. doi: 10.1371/journal.pgen.1005756 (PMC4711968; doi:10.1371/journal.pgen.1005756)
Supplement: S4 Table — (DOC) [file pgen.1005756.s011.doc]

**S4 Table. Comparison of minigene splicing data with ESR-dedicated bioinformatics predictions for *BRCA1* exon 6 variants.** The effect on splicing of 42 *BRCA1* exon 6 variants located outside the reference splice sites was previously determined in a pCR3.1-BRCA1-exons-5-7 minigene assay (WT exon inclusion level being estimated at 70 ±10%) [1]. Variants indicated in bold represent “artificial” SNVs generated by mutagenesis for research purposes, whereas variants in regular text are “natural” SNVs identified in patients [1]. The table shows the separation of the variants into 2 groups, according to the minigene results: variants that increased exon skipping (n=4) and those that did not (n=38, including 30 variants with no effect and 8 variants that increased exon inclusion, as indicated by the separating line). .*In silico* predictions of potential effects on splicing were conducted by using 3 newly developed ESR-dedicated approaches (ΔtESRseq, ΔHZ_EI_ and ΔΨ), as well as one prior method (EX-SKIP), as described under Materials and Methods. True and false calls (color codes indicated underneath the table) of exon-skipping events were determined by taking into account the following thresholds: -0.5 for ∆tESRseq, -20 for ∆HZ_EI_, -0.05 for ∆Ψ, and 1 for EX-SKIP. nd, not determined.

|  | *BRCA1* variant  (n=42) | Exon 6 inclusion (%) | New *in silico* approaches | | | Prior  *in silico* approach |
| --- | --- | --- | --- | --- | --- | --- |
|  |  |  | ∆tESRseq | ∆HZ_EI_ | ∆Ψ | EX-SKIP  (ESE/ESS) |
|  | WT | 70 | 0 | 0 | 0 | 1 |
| Variants that increased exon skipping  (n=4) | **c.224A>T** | 54 | -3.94 | -105.25 | -0.0035 | 0.83 |
|  | **c.230C>A** | 58 | -0.55 | -75.58 | -0.0139 | 0.97 |
|  | **c.230C>G** | 55 | -2.01 | -61.59 | -0.0153 | 0.97 |
|  | c.231G>T | 39 | -2.51 | -84.89 | -0.012 | 0.89 |
| Variants that did not increase exon skipping  (n=38) | c.216C>A | nd | 1.58 | 63.36 | 0.0011 | 1.05 |
|  | **c.224A>G** | 73 | -0.79 | -6.48 | 0.0011 | 1 |
|  | **c.225A>C** | 73 | -0.59 | -7.74 | 0.0003 | 0.94 |
|  | **c.225A>G** | 78 | -0.11 | -20.79 | -0.0023 | 0.94 |
|  | **c.225A>T** | 64 | -2.61 | -62.16 | -0.0039 | 0.89 |
|  | **c.226A>C** | 71 | 1.48 | 0.78 | 0.0023 | 0.94 |
|  | **c.226A>G** | 75 | -0.20 | 9.09 | -0.0028 | 0.93 |
|  | **c.226A>T** | 77 | 0.16 | -36.01 | 0.003 | 0.93 |
|  | **c.227G>A** | 67 | 0.00 | -22.58 | -0.0021 | 0.98 |
|  | **c.227G>C** | 79 | 1.89 | 26.08 | 0.002 | 1.08 |
|  | **c.227G>T** | 74 | 0.31 | -24.03 | -0.0102 | 0.96 |
|  | **c.229A>C** | 79 | 0.85 | 6.44 | -0.0056 | 0.90 |
|  | **c.230C>T** | 66 | -0.89 | -54.16 | -0.0076 | 0.96 |
|  | **c.231G>A** | 71 | 0.65 | 4.7 | -0.0039 | 1.08 |
|  | **c.231G>C** | 66 | -0.67 | -8.76 | -0.0041 | 0.98 |
|  | **c.232A>G** | 77 | 0.65 | -20.68 | 0.001 | 0.91 |
|  | **c.232A>T** | 75 | -0.27 | -48.57 | -0.3438 | 0.93 |
|  | **c.233G>A** | 67 | -0.91 | -39.94 | -0.0005 | 1 |
|  | **c.233G>C** | 78 | 0.08 | -27.97 | 0.0026 | 1.02 |
|  | **c.233G>T** | 65 | -1.79 | -72.41 | -0.0008 | 0.85 |
|  | **c.234A>C** | 70 | -1.59 | -5.4 | 0.0027 | 1.02 |
|  | **c.234A>G** | 65 | -1.71 | -18.26 | -0.0038 | 0.82 |
|  | **c.234A>T** | 78 | -0.98 | -52.39 | -0.0012 | 0.85 |
|  | **c.235T>A** | 80 | -0.02 | 24.96 | 0.0012 | 1.16 |
|  | **c.235T>G** | 66 | -0.17 | 21.8 | 0.003 | 1 |
|  | c.259T>G | nd | -0.93 | 0.75 | -0.0025 | 1.05 |
|  | c.266T>C | nd | -0.29 | 37.32 | 0.0015 | 1.12 |
|  | c.269T>C | nd | 0.11 | 38.02 | 0.0034 | 1.10 |
|  | c.286G>A | nd | -1.02 | -37.23 | -0.0117 | 1.03 |
|  | c.292G>C | nd | 1.74 | 29.47 | 0.0033 | 1.14 |
|  | **c.224A>C** | 85 | -1.61 | -13.89 | 0.0007 | 0.97 |
|  | **c.228T>A** | 83 | 2.42 | 55.65 | -0.0038 | 1.12 |
|  | **c.228T>C** | 82 | 0.30 | 21.3 | -0.0043 | 1.02 |
|  | **c.228T>G** | 85 | 2.47 | 39.72 | 0.0016 | 1.07 |
|  | **c.229A>G** | 86 | 0.35 | 0.92 | -0.0027 | 0.98 |
|  | **c.229A>T** | 81 | 2.17 | 3.97 | -0.0033 | 0.91 |
|  | **c.232A>C** | 85 | 0.94 | -18.44 | -0.0028 | 0.94 |
|  | **c.235T>C** | 82 | -0.22 | 24.44 | 0.0019 | 1.14 |
| **True**  **calls** | Positive | | 4 | 4 | 0 | 4 |
|  | Negative | | 25 | 25 | 37 | 18 |
|  | **Total** | | **29** | **29** | **37** | **22** |
| **False calls** | Positive | | 13 | 13 | 1 | 20 |
|  | Negative | | 0 | 0 | 4 | 0 |
|  | **Total** | | **13** | **13** | **5** | **20** |
| Sensitivity (%) | | | 100 | 100 | 0 | 100 |
| Specificity (%) | | | 66 | 66 | 97 | 47 |

| **True positive calls** | **True negative calls** | **False positive calls** | **False negative calls** |
| --- | --- | --- | --- |

1. Raponi M, Kralovicova J, Copson E, Divina P, Eccles D, Johnson P, et al. Prediction of single-nucleotide substitutions that result in exon skipping: identification of a splicing silencer in BRCA1 exon 6. Hum Mutat. 2011;32: 436–444. doi:10.1002/humu.21458
