# Supplementary material for: Exonic Splicing Mutations Are More Prevalent than Currently Estimated and Can Be Predicted by Using In Silico Tools
Source: PLoS Genet. 2016 Jan 13;12(1):e1005756. doi: 10.1371/journal.pgen.1005756 (PMC4711968; doi:10.1371/journal.pgen.1005756)
Supplement: S5 Table — (DOC) [file pgen.1005756.s012.doc]

**S5 Table. Comparison of minigene splicing data with ESR-dedicated bioinformatics predictions for *CFTR* exon 12 variants.** The effect on splicing of 41 *CFTR* exon 12 variants located outside the reference splice sites was previously determined in a WTB-CFTR-exon 12 minigene assay [1,2]. Variants indicated in bold represent “artificial” SNVs generated by mutagenesis for research purposes, whereas variants in regular text are “natural” SNVs identified in patients [1,2]. The table shows a separation of the variants into 2 groups according to the minigene results: variants that increased exon skipping (n=22) and those that did not (n=19, including one variant with not effect and 18 that increased exon inclusion, as indicated by the separating line). *In silico* predictions of potential effects on splicing were conducted by using 3 newly developed ESR-dedicated approaches (ΔtESRseq, ΔHZ_EI_ and ΔΨ,) as well as one prior method (EX-SKIP). True and false calls (color codes indicated underneath the table) of exon-skipping events were determined by taking into account the following thresholds: -0.5 for ∆tESRseq, -20 for ∆HZ_EI_, -0.05 for ∆Ψ, and 1 for EX-SKIP.

|  | CFTR variant  (n=41) | | Exon 12 inclusion (~%) | | New in silico approaches | | | | Prior  in silico approach | |
| --- | --- | --- | --- | --- | --- | --- | --- | --- | --- | --- |
|  |  |  |  |  | ∆tESRseq | ∆HZei | | ∆PSI | EX-SKIP (ESE/ESS) | |
|  | WT | | 80 | | 0 | 0 | | 0 | 1 | |
| Variants that increased exon skipping  (n=22) | **c.1691A>T** | | 8 | | -1.46 | -95.57 | | -0.0362 | 0.76 | |
|  | **c.1692A>T** | | 60 | | -0.55 | -8.68 | | 0.0113 | 0.84 | |
|  | **c.1693G>A** | | 10 | | -2.19 | -88.10 | | -0.053 | 0.75 | |
|  | **c.1693G>C** | | 10 | | -1.15 | -62.02 | | -0.0149 | 0.86 | |
|  | **c.1693G>T** | | 5 | | -2.65 | -110.14 | | -0.0052 | 0.76 | |
|  | **c.1694A>T** | | 50 | | -1.00 | -81.07 | | -0.0273 | 0.76 | |
|  | c.1694A>G | | 35 | | -1.97 | -64.05 | | -0.0475 | 0.8 | |
|  | **c.1696G>A** | | 5 | | -0.24 | -79.13 | | -0.0116 | 0.93 | |
|  | **c.1696G>C** | | 45 | | 0.42 | -15.10 | | -0.0089 | 1.07 | |
|  | **c.1697C>T** | | 5 | | -0.37 | -70.25 | | -0.0038 | 0.87 | |
|  | **c.1697C>G** | | 40 | | -0.81 | -30.13 | | -0.0169 | 0.84 | |
|  | **c.1698T>A** | | 40 | | -0.26 | 21.20 | | -0.0142 | 1.05 | |
|  | **c.1704G>A** | | 25 | | -1.30 | -33.09 | | -0.0311 | 0.99 | |
|  | **c.1719T>C** | | 5 | | -1.34 | -16.88 | | 0.0068 | 1.03 | |
|  | **c.1726G>A** | | 0 | | -3.85 | -78.58 | | -0.0621 | 0.93 | |
|  | **c.1727G>A** | | 5 | | -0.37 | -49.59 | | -0.0091 | 0.99 | |
|  | c.1727G>C | | 7 | | -1.97 | -52.42 | | -0.011 | 0.92 | |
|  | **c.1728A>T** | | 10 | | -1.85 | -56.16 | | -0.0471 | 0.84 | |
|  | **c.1728A>G** | | 10 | | -2.61 | -60.83 | | -0.0376 | 0.83 | |
|  | **c.1729T>G** | | 10 | | 0.96 | 46.29 | | 0.0085 | 1.08 | |
|  | **c.1731C>T** | | 15 | | -0.41 | -19.71 | | 0.0126 | 0.94 | |
|  | **c.1731C>G** | | 5 | | -2.09 | -16.37 | | -0.7787 | 0.95 | |
| Variants that did not increase exon skipping  (n=19) | **c.1729T>A** | | 75 | | 0.60 | 16.12 | | -0.007 | 1.11 | |
|  | **c.1691A>G** | | 98 | | 0.35 | -9.28 | | -0.0138 | 0.99 | |
|  | **c.1692A>G** | | 95 | | 0.90 | 2.39 | | -0.0099 | 0.92 | |
|  | **c.1695T>A** | | 95 | | 1.03 | 44.54 | | 0.0123 | 1.07 | |
|  | **c.1695T>C** | | 95 | | 1.98 | -31.03 | | 0.0122 | 0.93 | |
|  | **c.1695T>G** | | 90 | | -0.94 | -55.12 | | -0.0352 | 0.94 | |
|  | **c.1698T>C** | | 95 | | 0.50 | 25.57 | | 0.0096 | 0.98 | |
|  | **c.1698T>G** | | 95 | | 1.73 | 8.19 | | 0.0267 | 0.99 | |
|  | **c.1701T>C** | | 90 | | 0.58 | 46.34 | | 0.0213 | 1.07 | |
|  | **c.1707T>C** | | 95 | | 0.95 | 86.92 | | 0.0255 | 1.28 | |
|  | **c.1713A>G** | | 100 | | 2.52 | 62.06 | | 0.0478 | 1.08 | |
|  | **c.1716C>T** | | 100 | | -0.69 | -5.09 | | -0.0077 | 0.92 | |
|  | **c.1719T>A** | | 90 | | -0.35 | 43.34 | | 0.0194 | 1.05 | |
|  | **c.1719T>G** | | 100 | | 1.10 | 66.23 | | 0.0289 | 1 | |
|  | **c.1722T>A** | | 100 | | 0.27 | 47.54 | | 0.0226 | 1.13 | |
|  | **c.1722T>C** | | 90 | | 0.36 | 12.36 | | 0.0032 | 1.11 | |
|  | **c.1722T>G** | | 90 | | 2.35 | 77.53 | | 0.0247 | 1.01 | |
|  | c.1730A>T | | 95 | | -0.23 | -15.24 | | -0.0158 | 0.93 | |
|  | **c.1730A>G** | | 98 | | 0.03 | 17.40 | | -0.0018 | 0.96 | |
| **True**  **calls** | Positive | | | | 15 | 15 | | 2 | 18 | |
|  | Negative | | | | 17 | 17 | | 19 | 10 | |
|  | **Total** | | | | **32** | **32** | | **21** | **28** | |
| **False calls** | Positive | | | | 2 | 2 | | 0 | 9 | |
|  | Negative | | | | 7 | 7 | | 20 | 4 | |
|  | **Total** | | | | **9** | **9** | | **20** | **13** | |
| Sensitivity (%) | | | | | 68 | 68 | | 9 | 82 | |
| Specificity (%) | | | | | 89 | 89 | | 100 | 53 | |
|  |  | |  | |  |  | |  |  | |
| **True positive calls** | | **True negative calls** | | **False positive calls** | | | **False negative calls** | | |  |

1. Pagani F, Stuani C, Tzetis M, Kanavakis E, Efthymiadou A, Doudounakis S, et al. New type of disease causing mutations: the example of the composite exonic regulatory elements of splicing in CFTR exon 12. Hum Mol Genet. 2003;12: 1111–1120.

2. Pagani F, Raponi M, Baralle FE. Synonymous mutations in CFTR exon 12 affect splicing and are not neutral in evolution. Proc Natl Acad Sci U S A. 2005;102: 6368–6372. doi:10.1073/pnas.0502288102
