# Supplementary material for: Exonic Splicing Mutations Are More Prevalent than Currently Estimated and Can Be Predicted by Using In Silico Tools
Source: PLoS Genet. 2016 Jan 13;12(1):e1005756. doi: 10.1371/journal.pgen.1005756 (PMC4711968; doi:10.1371/journal.pgen.1005756)
Supplement: S6 Table — (DOC) [file pgen.1005756.s013.doc]

**S6 Table. Comparison of minigene splicing data with ESR-dedicated bioinformatics predictions for *NF1* exon 37 variants.** The effect on splicing of 24 *NF1* exon 37 variants located outside the reference splice sites was previously determined in a PTB–NF1-exons-34-38 minigene assay [1]**.** Variants indicated in bold represent “artificial” SNVs generated by mutagenesis for research purposes, whereas variants in regular text are “natural” SNVs identified in patients [1]. The table shows the separation of the variants into 2 groups according to the minigene results: variants that increased exon skipping (n=6) and those with no effect (n=18). Informations about exon inclusion levels were not available in [1]. *In silico* predictions of potential effects on splicing were conducted by using 3 newly developed ESR-dedicated approaches (ΔtESRseq, ΔHZ_EI_ and ΔΨ), as well as one prior method, (EX-SKIP), as described under Materials and Methods. True and false calls (color code indicated underneath the table) of exon-skipping events were determined by taking into account the following thresholds: -0.5 for ∆tESRseq, -20 for ∆HZ_EI_, -0.05 for ∆Ψ, and 1 for EX-SKIP. .

|  | NF1 variant  (n=24) | New *in silico* approaches | | | Prior  *in silico* approach |
| --- | --- | --- | --- | --- | --- |
|  |  | ∆tESRseq | ∆HZei | ∆Ψ | EX-SKIP (ESE/ESS) |
| Variants that increased  exon skipping  (n=6) | c.6792C>A | -1.71 | -89.1 | -0.6894 | 0.92 |
|  | c.6792C>G | -1.11 | -44.1 | -0.6879 | 0.97 |
|  | **c.6792C>T** | -1.80 | -90.5 | -0.0045 | 0.80 |
|  | **c.6795C>A** | 0.30 | -34.5 | 0.0013 | 0.94 |
|  | **c.6795C>G** | 1.81 | -1.37 | 0.0098 | 1.03 |
|  | **c.6795C>T** | -1.57 | -76.1 | -0.0085 | 0.87 |
| Variants that did not increase exon skipping  (n=18) | **c.6790T>A** | 0.78 | -2.7 | 0.0017 | 0.97 |
|  | **c.6790T>C** | 1.22 | 16.07 | -0.0065 | 0.89 |
|  | **c.6790T>G** | 2.36 | 45.87 | 0.0317 | 1.03 |
|  | **c.6791A>C** | 1.13 | 24.78 | 0.0168 | 0.94 |
|  | **c.6791A>G** | 0.83 | 7.38 | 0.0109 | 0.94 |
|  | **c.6791A>T** | 0.52 | -25.1 | 0.0091 | 0.94 |
|  | **c.6793A>C** | 0.08 | -23.1 | -0.0042 | 0.92 |
|  | **c.6793A>G** | 1.41 | 8.57 | 0.0159 | 0.97 |
|  | **c.6793A>T** | 0.30 | -23.5 | -0.0072 | 0.94 |
|  | **c.6794A>C** | -0.58 | -1.22 | -0.0081 | 0.97 |
|  | **c.6794A>G** | -0.21 | 18.26 | -0.0031 | 0.97 |
|  | **c.6794A>T** | -0.10 | -18.7 | 0.0073 | 0.97 |
|  | **c.6796A>C** | 1.16 | 9.67 | 0.0177 | 0.97 |
|  | **c.6796A>G** | 1.93 | 2.43 | 0.0367 | 0.97 |
|  | **c.6796A>T** | 0.87 | -19.6 | 0.009 | 0.97 |
|  | **c.6797G>A** | 0.57 | 12.28 | 0.0228 | 1.03 |
|  | **c.6797G>C** | 0.93 | -13.9 | -0.0199 | 0.94 |
|  | **c.6797G>T** | 0.75 | -25.6 | -0.0118 | 0.92 |
| **True**  **calls** | Positive | 4 | 5 | 2 | 5 |
|  | Negative | 17 | 14 | 18 | 2 |
|  | **Total** | **21** | **19** | **20** | **7** |
| **False**  **calls** | Positive | 1 | 4 | 0 | 16 |
|  | Negative | 2 | 1 | 4 | 1 |
|  | **Total** | **3** | **5** | **4** | **17** |
| Sensitivity (%) | | 67 | 83 | 33 | 83 |
| Specificity (%) | | 94 | 78 | 100 | 11 |

| **True positive calls** | **True negative calls** | **False positive calls** | **False negative calls** |
| --- | --- | --- | --- |

1. Baralle M, Skoko N, Knezevich A, De Conti L, Motti D, Bhuvanagiri M, et al. NF1 mRNA biogenesis: effect of the genomic milieu in splicing regulation of the NF1 exon 37 region. FEBS Lett. 2006;580: 4449–4456. doi:10.1016/j.febslet.2006.07.018
