# Supplementary material for: Exonic Splicing Mutations Are More Prevalent than Currently Estimated and Can Be Predicted by Using In Silico Tools
Source: PLoS Genet. 2016 Jan 13;12(1):e1005756. doi: 10.1371/journal.pgen.1005756 (PMC4711968; doi:10.1371/journal.pgen.1005756)
Supplement: S7 Table — (DOC) [file pgen.1005756.s014.doc]

**S7 Table.** **Comparative statistical analysis of the predictive power of four ESR-dedicated bioinformatics approaches by using five independent datasets.** The experimental datasets indicated on the left column (*MLH1* exon 10, *BRCA2* exon 7, *BRCA1* exon 6, *CFTR* exon 12 and *NF1* exon 37), all derived from minigene assays (this study and [1–5]), were first compared with results obtained with 3 newly developed ESR-dedicated *in silico* prediction tools (ΔtESRseq, ΔHZ_EI_ and ΔΨ) and one prior method (EX-SKIP) as shown in Tables S2, S3, S4, S5 and S6. Statistical analyses (Student’s t-test, ANOVA test and Pearson correlation coefficient, as indicated) were performed as described under Materials and Methods. Results are expressed as p-values. Pearson’s correlation coefficient values [r] are indicated between brackets. The number of variants (n=) taken into consideration in each statistical analysis is highlighted in grey. n/a, not applicable; * indicates that the number of variants taken into consideration is inferior to that indicated in the left column, as explained in S9 Table.

|  | New *in silico* approaches | | | | | | | | | Prior *in silico* approach | | |
| --- | --- | --- | --- | --- | --- | --- | --- | --- | --- | --- | --- | --- |
| Experimental datasets | ∆tESRseq | | | ∆HZ_EI_ | | | ∆Ψ | | | EX-SKIP  (ESE/ESS) | | |
|  | t-test | ANOVA | Pearson | t-test | ANOVA | Pearson | t-test | ANOVA | Pearson | t-test | ANOVA | Pearson |
| *MLH1* exon10 | 0.01 | 0.03 | 0.001 [0.72] | 0.11 | 0.15 | 0.004 [0.67] | 0.42 | 0.59 | 0.93 [-0.02] | 0.22 | 0.26 | 0.02 [0.55] |
| n=15 | 15 | 15 | 15 | 15 | 15 | 15 | 15 | 15 | 15 | 15 | 15 | 15 |
| *BRCA2* exon 7 | 3.5 e^-6^ | n/a | 1.1 e^-6^ [0.74] | 5.7 e-6 | n/a | 0.9 e^-3^ [0.55] | 0.56 | n/a | 0.15 [0.28] | 0.02 | n/a | 0.008 [0.45] |
| n=32 | 32 | - | 32 | 32 | - | 32 | 27* | - | 27* | 32 | - | 32 |
| *BRCA1* exon 6 | 0.04 | 0.0004 | 2.7 e^-6^ [0.68] | 0.0005 | 4.06 e^-5^ | 1.2 e^-6^ [0.7] | 0.92 | 0.87 | 0.99 [0.0007] | 0.1 | 0.15 | 0.01 [0.4] |
| n=42 | 42 | 42 | 36* | 42 | 42 | 36* | 42 | 42 | 36* | 42 | 42 | 36* |
| *CFTR* exon 12 | 7.1 e^-7^ | n/a | 1.1 e^-7^ [0.71] | 9.8 e^-7^ | n/a | 2.08 e^-7^ [0.7] | 0.09 | n/a | 0.05 [0.3] | 0.0002 | n/a | 0.0009 [0.49] |
| n=41 | 41 | - | 41 | 41 | - | 41 | 41 | - | 41 | 41 | - | 41 |
| *NF1* exon 37 | 0.05 | n/a | n/a | 0.01 | n/a | n/a | 0.16 | n/a | n/a | 0.31 | n/a | n/a |
| n=24 | 24 | - | - | 24 | - | - | 24 | - | - | 24 | - | - |

1. Di Giacomo D, Gaildrat P, Abuli A, Abdat J, Frébourg T, Tosi M, et al. Functional analysis of a large set of BRCA2 exon 7 variants highlights the predictive value of hexamer scores in detecting alterations of exonic splicing regulatory elements. Hum Mutat. 2013;34: 1547–1557. doi:10.1002/humu.22428

2. Raponi M, Kralovicova J, Copson E, Divina P, Eccles D, Johnson P, et al. Prediction of single-nucleotide substitutions that result in exon skipping: identification of a splicing silencer in BRCA1 exon 6. Hum Mutat. 2011;32: 436–444. doi:10.1002/humu.21458

3. Pagani F, Stuani C, Tzetis M, Kanavakis E, Efthymiadou A, Doudounakis S, et al. New type of disease causing mutations: the example of the composite exonic regulatory elements of splicing in CFTR exon 12. Hum Mol Genet. 2003;12: 1111–1120.

4. Pagani F, Raponi M, Baralle FE. Synonymous mutations in CFTR exon 12 affect splicing and are not neutral in evolution. Proc Natl Acad Sci U S A. 2005;102: 6368–6372. doi:10.1073/pnas.0502288102

5. Baralle M, Skoko N, Knezevich A, De Conti L, Motti D, Bhuvanagiri M, et al. NF1 mRNA biogenesis: effect of the genomic milieu in splicing regulation of the NF1 exon 37 region. FEBS Lett. 2006;580: 4449–4456. doi:10.1016/j.febslet.2006.07.018
