# Supplementary material for: Exonic Splicing Mutations Are More Prevalent than Currently Estimated and Can Be Predicted by Using In Silico Tools
Source: PLoS Genet. 2016 Jan 13;12(1):e1005756. doi: 10.1371/journal.pgen.1005756 (PMC4711968; doi:10.1371/journal.pgen.1005756)
Supplement: S8 Table — (DOC) [file pgen.1005756.s015.doc]

**S8 Table.** **Comparative analysis of** **the** **sensitivity and specificity of ESR-dedicated bioinformatics approaches in predicting exon-skipping mutations by using five independent datasets.** The experimental datasets indicated on the left column (*MLH1* exon 10, *BRCA2* exon 7, *BRCA1* exon 6, *CFTR* exon 12 and *NF1* exon 37), all derived from minigene assays (this study and [1–5]), were first compared with results obtained with 3 newly developed ESR-dedicated *in silico* prediction tools (ΔtESRseq, ΔHZ_EI_ and ΔΨ) and one prior method (EX-SKIP) as shown in Tables S2, S3, S4, S5 and S6. True and false calls of exon-skipping events were determined by taking into account the following thresholds: -0.5 for ∆tESRseq, -20 for ∆HZ_EI_, -0.05 for ∆Ψ, and 1 for EX-SKIP. Sensitivity and specificity were calculated as described under Materials and Methods. n=, number of variants; * indicates that number of variants taken into consideration is inferior to that indicated in the left column, as explained in S9 Table.

|  | New *in silico* approaches | | | | | | Previous *in silico* approach | |
| --- | --- | --- | --- | --- | --- | --- | --- | --- |
|  | ∆tESRseq | | ∆HZ_EI_ | | ∆Ψ | | EX-SKIP  (ESE/ESS) | |
|  | Sensitivity  (%) | Specificity  (%) | Sensitivity  (%) | Specificity  (%) | Sensitivity  (%) | Specificity  (%) | Sensitivity  (%) | Specificity  (%) |
| *MLH1* exon10 (n=15) | 86 | 88 | 57 | 63 | 29 | 88 | 43 | 75 |
| *BRCA2* exon 7 (n=32) | 100 | 86 | 91 | 76 | 18* | 94* | 64 | 62 |
| *BRCA1* exon 6 (n=42) | 100 | 66 | 100 | 66 | 0 | 97 | 100 | 47 |
| *CFTR* exon 12 (n=41) | 68 | 89 | 68 | 89 | 9 | 100 | 82 | 53 |
| *NF1* exon 37 (n=24) | 67 | 94 | 83 | 78 | 33 | 100 | 83 | 11 |

1. Di Giacomo D, Gaildrat P, Abuli A, Abdat J, Frébourg T, Tosi M, et al. Functional analysis of a large set of BRCA2 exon 7 variants highlights the predictive value of hexamer scores in detecting alterations of exonic splicing regulatory elements. Hum Mutat. 2013;34: 1547–1557. doi:10.1002/humu.22428

2. Raponi M, Kralovicova J, Copson E, Divina P, Eccles D, Johnson P, et al. Prediction of single-nucleotide substitutions that result in exon skipping: identification of a splicing silencer in BRCA1 exon 6. Hum Mutat. 2011;32: 436–444. doi:10.1002/humu.21458

3. Pagani F, Stuani C, Tzetis M, Kanavakis E, Efthymiadou A, Doudounakis S, et al. New type of disease causing mutations: the example of the composite exonic regulatory elements of splicing in CFTR exon 12. Hum Mol Genet. 2003;12: 1111–1120.

4. Pagani F, Raponi M, Baralle FE. Synonymous mutations in CFTR exon 12 affect splicing and are not neutral in evolution. Proc Natl Acad Sci U S A. 2005;102: 6368–6372. doi:10.1073/pnas.0502288102

5. Baralle M, Skoko N, Knezevich A, De Conti L, Motti D, Bhuvanagiri M, et al. NF1 mRNA biogenesis: effect of the genomic milieu in splicing regulation of the NF1 exon 37 region. FEBS Lett. 2006;580: 4449–4456. doi:10.1016/j.febslet.2006.07.018
