# Supplementary material for: Exonic Splicing Mutations Are More Prevalent than Currently Estimated and Can Be Predicted by Using In Silico Tools
Source: PLoS Genet. 2016 Jan 13;12(1):e1005756. doi: 10.1371/journal.pgen.1005756 (PMC4711968; doi:10.1371/journal.pgen.1005756)
Supplement: S9 Table — (DOC) [file pgen.1005756.s016.doc]

**S9 Table. Explanations for the exceptions in the total number of variants taken into account in statistical analyses described in Tables S3, S7 and S8.** Grey and white empty boxes indicate *in silico* approaches in which a dataset exception was applied or not, respectively. Number of variants is indicated for each case (n=).

| Experimental datasets | *In silico* approaches | | | | Statistical methods | Statistical analysis exceptions | Explanations | Tables |
| --- | --- | --- | --- | --- | --- | --- | --- | --- |
|  | ∆tESRseq | ∆HZ_EI_ | ∆Ψ | EX-SKIP |  |  |  |  |
| *BRCA2*  exon 7  (n=32) |  |  |  |  | ANOVA | “not applicable” (n/a) | *BRCA2* exon7 dataset cannot be separated into 3 groups (separated into 2 groups only) | S7 |
|  |  |  |  |  | t-test and Pearson | n=27 instead of n=32 | ∆Ψ values cannot be calculated for del/ins variants (n=5) | S3, S7 and S8 |
| *BRCA1*  exon 6  (n=42) |  |  |  |  | Pearson | n=36 instead of n=42 | Levels of exon inclusion (%) are not available  for 6 of the 42 *BRCA1* exon 6 variants | S7 |
| *CFTR*  exon12  (n=41) |  |  |  |  | ANOVA | “not applicable” (n/a) | *CFTR* exon12 dataset can be separated into 3 groups  but one of the groups contains 1 variant only | S7 |
| *NF1*  exon 37  (n=24) |  |  |  |  | ANOVA | “not applicable” (n/a) | *NF1* exon 37 dataset cannot be separated into 3 groups (separated into 2 groups only) | S7 |
|  |  |  |  |  | Pearson | “not applicable” (n/a) | Levels of exon inclusion are not available  for any of the 24 *NF1* exon 37 variants | S7 |
